# Functions of prolyl hydroxylation in elastin

**DOI:** 10.1016/j.jbc.2026.111323

**Published:** 2026-02-26

**Authors:** Chengeng Yang, Christian E.H. Schmelzer, Anna Tarakanova

**Affiliations:** 1 Department of Biomedical Engineering, University of Connecticut, Storrs, Connecticut, USA; 2 Department of Biological and Macromolecular Materials, Fraunhofer Institute for Microstructure of Materials and Systems IMWS, Halle (Saale), Germany; 3 Institute of Pharmacy, Faculty of Natural Sciences I, Martin Luther University Halle-Wittenberg, Halle (Saale), Germany; 4 School of Mechanical, Aerospace, and Manufacturing Engineering, University of Connecticut, Storrs, Connecticut, USA

**Keywords:** tropoelastin, hydroxyproline, prolyl hydroxylation, hydration, protein dynamics, molecular dynamics

## Abstract

Elastin is a key protein responsible for elasticity, resilience, and deformability of tissues. Elastin is subject to an understudied posttranslational modification, prolyl hydroxylation, where a hydroxyl group replaces a hydrogen atom at C-γ in proline residues during assembly. Recent experimental studies suggest elastin-like peptides with hydroxyproline modifications are more resistant to enzymatic digestion and subject to abnormal assembly. We hypothesize that hydroxylation modulates protein-solvent interactions, thereby altering elastin behavior. To test our hypothesis, we build representative models with and without prolyl hydroxylation and perform extensive molecular dynamics simulations. Our findings suggest that hydroxyproline increases hydrogen bonding with water by an average of 135% compared to proline, which reduces the local configurational space, thereby negatively impacting elastin’s global dynamics, essential for its biological functions. This modification may potentially protect the molecule from targeted degradation and modulate canonical hierarchical assembly. In addition, our study provides design insights for engineered elastin-based materials through fine-tuning of hydroxyproline content.

Connective tissues and organs of high tensile strength, such as the lung, heart and skin, extensively express elastin (1). Elastin provides elasticity and resilience and contributes to the mechanical deformability of these tissues and organs. On this basis, elastin-based materials have been widely applied in various fields such as tissue regeneration and drug delivery (2, 3, 4). The process of elastic fiber formation, elastogenesis (5, 6), is realized through the hierarchical assembly of the elastin monomer, tropoelastin, which is secreted from and attached to the surface of specific cell types, such as vascular smooth muscle cells, followed by spatial rearrangement and self-assembly through coacervation resulting in elastin multimers (5). These multimers further undergo lysyl oxidase-mediated intermolecular and/or intramolecular cross-linking while depositing on fibrillin microfibrils, eventually forming mature elastic fibers (5, 6). There are several posttranslational modifications (PTMs) in elastin, including cross-linking and prolyl hydroxylation. Cross-linking is a prerequisite for mature elastic fiber formation and a likely contributor to mechanical properties. The role of prolyl hydroxylation and its underlying molecular mechanisms, however, have not yet been revealed. This modification, occurring before tropoelastin secretion, is mediated by prolyl 4-hydroxylase, which replaces a hydrogen atom at the C-γ position of proline residues (Pro) with a hydroxyl group, rendering a modification on Pro, (2S,4R)-4-hydroxyproline (Hyp) (7, 8, 9, 10). The schematic drawing of elastogenesis featuring prolyl hydroxylation is shown in Figure 1.

**Figure 1:**
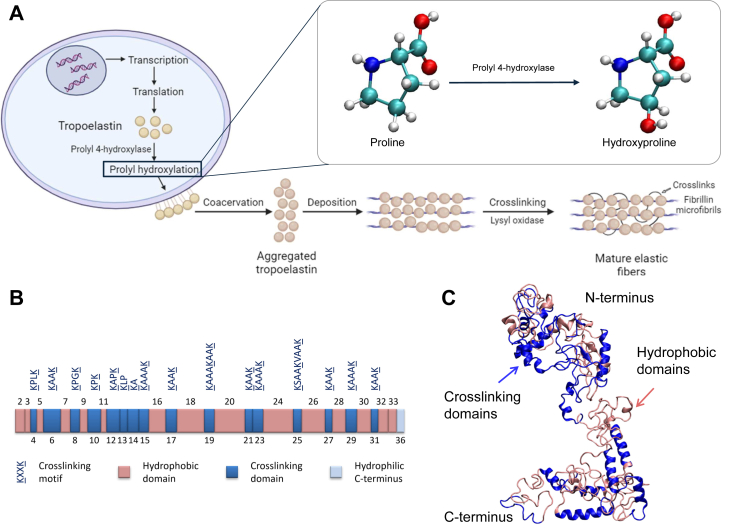
**Hierarchical elastic fiber assembly and tropoelastin domain arrangement.***A*, schematic drawing of elastogenesis, including transcription, protein translation, tropoelastin secretion, coacervation of tropoelastin into multimers, cross-linking by lysyl oxidase and deposition onto fibrillin microfibrils to form mature fibrils. The inset features prolyl hydroxylation. *B,* tropoelastin domain arrangement and (*C*) tropoelastin cartoon structure, with hydrophobic domains highlighted in *red* and cross-linking domains highlighted in *blue*.

The observation of prolyl hydroxylation in native elastin *via* amino acid analyses had historically been misconstrued as an artifact induced by collagen contamination during sample extraction (7, 11). However, recent studies of elastin hydrolysates using mass spectrometry (MS) have provided molecular-level evidence that mature elastin indeed contains Hyp residues. Peptide motifs are identified where prolyl hydroxylation occurs specifically in human elastin samples, namely Gly-Yaa-Pro-Gly and Gly-Yaa-Zaa-Pro-Gly (Yaa and Zaa can be any amino acid except proline) (7), suggesting specific sequence patterns involved in this modification. These motifs often contain Hyp residues; however, hydroxylation is partial, meaning that not all motifs are hydroxylated simultaneously in a given molecule. The hydroxylation degree of proline residues varies across different proline positions, different isoforms, different tissue types, and different species (7, 12, 13, 14, 15). For example, in samples collected from skin, aorta, cartilage and intervertebral disc (IVD), Pro190 is more than 70% hydroxylated, while Pro615 is less than 50% hydroxylated in human elastin samples (7). Both Pro positions are determined to be more hydroxylated in elastin samples from human IVD, compared to samples taken from skin, aorta, and ear cartilage. Pro190 and Pro615 are also found to be hydroxylated in elastin samples from other species, with various hydroxylation degrees. For instance, Pro190 is less hydroxylated in chicken elastin samples compared to its counterparts in human, pig, and cow elastin samples. Interestingly, age is not a deterministic factor in prolyl hydroxylation, possibly because this PTM occurs in the endoplasmic reticulum immediately after tropoelastin is translated from mRNA, and matured elastin exhibits little to no turnover throughout life (16, 17).

As the fourth most prevalent amino acid in human elastin, proline engages in the maintenance of structural heterogeneity in elastin and modulation of backbone flexibility (7). Experimental studies have found a preference for polyproline II (PPII) helix formation and a rise in the transition temperature associated with the coacervation process in elastogenesis, where tropoelastin aggregates and separates into two phases, in Hyp-containing elastin-like polypeptides (ELPs), which may contribute to a reduced tendency of Hyp-containing ELPs to form linear elastic fibers (8). Damped enzymatic protease digestion of Hyp-modified domain 18 of tropoelastin has also been identified (8). Despite these intriguing findings based on experimental studies of ELPs, the role prolyl hydroxylation plays in tuning elastin functionality is lacking.

Based on the key differences between Hyp and Pro, namely, hydroxyl group *versus* hydrogen atom connected to the C-γ position, we hypothesize that Hyp may leverage its boosted hydrophilicity stemming from the hydroxyl group to enhance protein-solvent interactions. This in turn would lead to alterations in protein dynamics and thus influence the susceptibility toward enzymatic degradation and canonical behaviors of hierarchical assembly. To investigate this hypothesis, we perform extensive molecular dynamics (MD) simulations on human elastin models—namely peptides, domains and the full-length monomer—with experimentally informed prolyl hydroxylation patterns, allowing us to examine molecular interactions at the atomic level. Our study provides insights into the functional roles of prolyl hydroxylation in elastin at the nanoscale.

## Results

### Prolyl hydroxylation induces heterogeneity in representative structural ensembles of elastin

**Figure 2:**
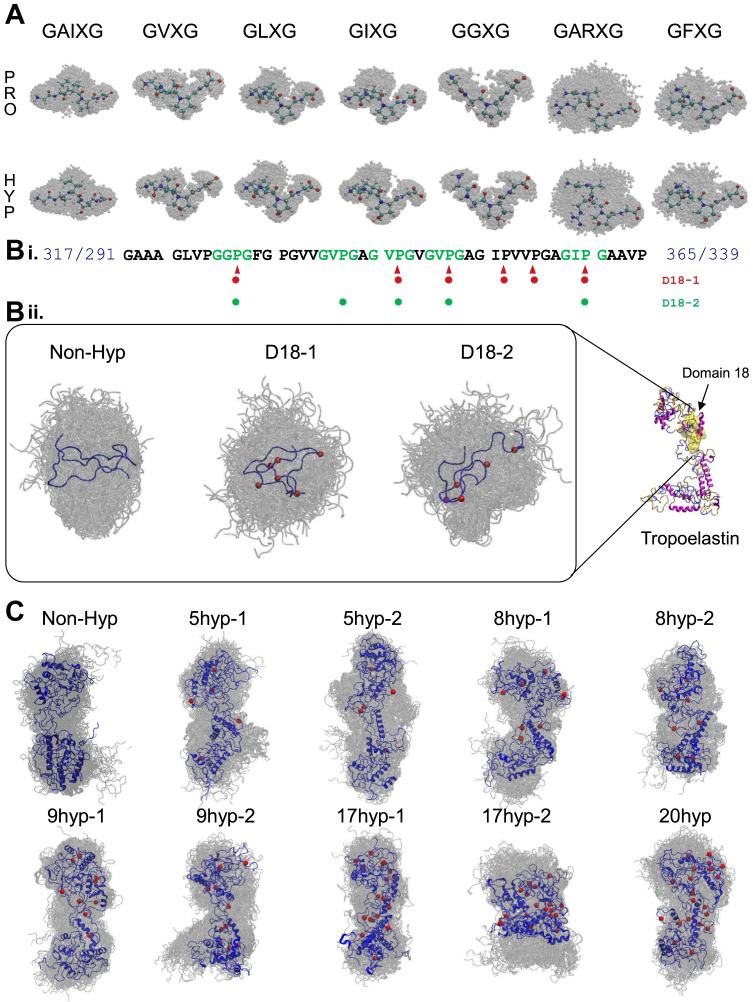
**Elastin models considered in this study.***A*, cartoon representations of representative structural ensembles of the motif models, GAIXG, GVXG, GLXG, GIXG, GGXG, GARXG, and GFXG, where X is Pro (*top row*) or Hyp (*bottom row*). The most representative structure for each condition is highlighted. (*B*, i) prolyl hydroxylation maps of the domain 18 models, Non-Hyp, D18-1, and D18-2. (ii) cartoon representations of representative structure ensembles in the domain 18 models, Non-Hyp, D18-1, and D18-2. The most representative structure for each condition is highlighted in *blue*, and the positions of hydroxyproline residues are annotated by *red* beads. *C*, cartoon representations of representative structural ensembles of the full-length tropoelastin models, Non-Hyp, 5hyp-1, 5hyp-2, 8hyp-1, 8hyp-2, 9hyp-1, 9hyp-2, 17hyp-1, 17hyp-2, and 20hyp. The most representative structure for each condition is highlighted in *blue*, and the positions of hydroxyproline residues are annotated by *red* beads. HYP, hydroxylated proline

**Figure 3:**
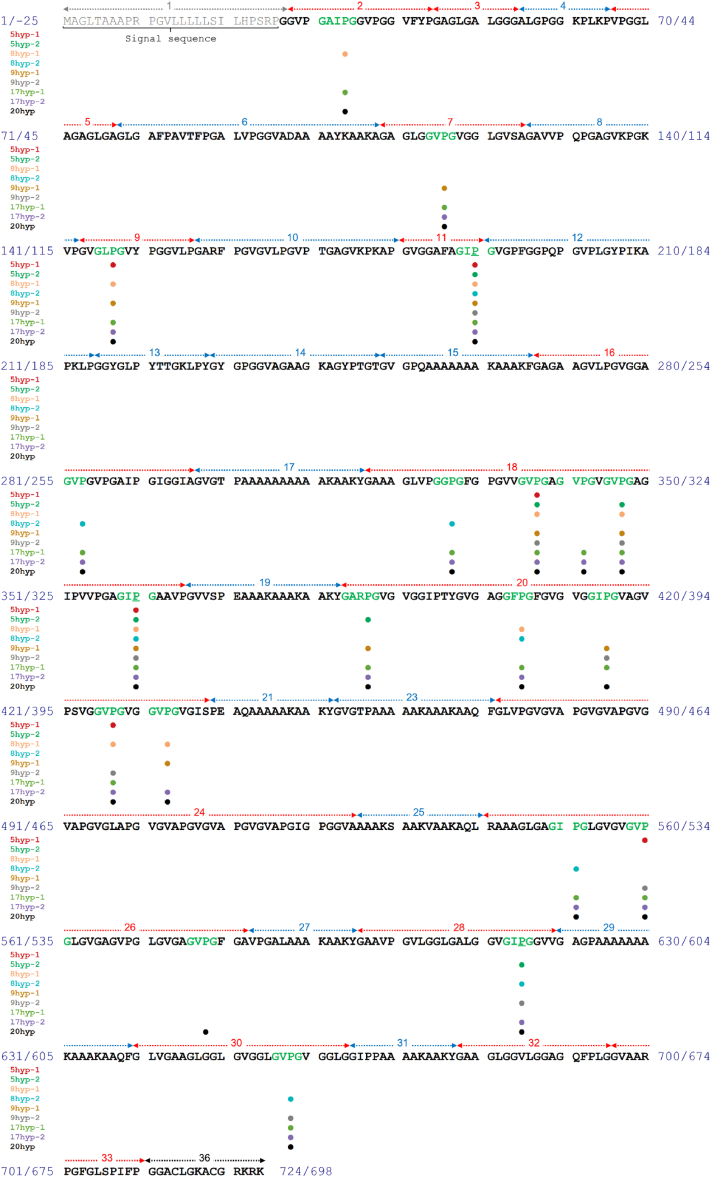
**Prolyl hydroxylation map of the full-length tropoelastin molecule based on high resolution mass spectrometric data of isolated and proteolyzed human elastin.***Filled circles* in different colors show proline residues hydroxylated in models Non-Hyp, 5hyp-1, 5hyp-2, 8hyp-1, 8hyp-2, 9hyp-1, 9hyp-2, 17hyp-1, 17hyp-2, and 20hyp. Designated Hyp sites (190, 360, and 615) are underlined. HYP, hydroxylated proline

We find that the introduction of hydroxyproline results in dissimilar structural ensembles in the models. The structural ensembles of nonhydroxylated and hydroxylated models reflecting representative structures from all clusters *via* the Jarvis Patrick clustering method and highlighting the representative structure from the most populated cluster (see Experimental procedures for further details) are shown in Figure 2, *A* and *B* (ii), and C, for the motif models, domain 18 models and the full-length tropoelastin models, respectively. Hydroxylation sites in full-length models are selected based on experimental data. Prolyl hydroxylation maps of the full-length tropoelastin based on high resolution mass spectrometric data of isolated and proteolyzed human elastin are presented in Figure 3. Selected prolyl hydroxylation sites in the full-length models are summarized in Table 1.

**Table 1:** Summary of prolyl hydroxylation sites in each of the full-length tropoelastin models

Model name	Designated Hyp sites	Randomly selected Hyp sites
Non-Hyp	N/A	N/A
5hyp-1	190, 360	147, 427, 560
5hyp-2	190, 360, 615	347, 387
8hyp-1	190, 360	34, 147, 347, 405, 427, 433
8hyp-2	190, 360, 615	283, 337, 405, 551, 658
9hyp-1	190, 360	116, 147, 327, 347, 387, 415, 433
9hyp-2	190, 360, 615	327, 347, 415, 427, 560, 658
17hyp-1	190, 360	34, 116, 147, 283, 327, 337, 342, 347, 387, 405, 415, 427, 551, 560, 658
17hyp-2	190, 360, 615	116, 147, 283, 327, 337, 342, 347, 387, 405, 427, 433, 551, 560, 658
20hyp (overhydroxylation)	All potential sites	All potential sites

Deviations in intramolecular atom-wise distance distributions among the representative ensembles of the motif models measured by distance maps are generally subtle (Fig. S1), such as in GAIPG *versus* GAIHypG, GLPG *versus* GLHypG, GIPG *versus* GIHypG, and GGPG *versus* GGHypG. But the deviations between GVPG *versus* GVHypG (especially in residue V), GARPG *versus* GARHypG (especially in residue R), as well as between GFPG *versus* GFHypG (especially in residue F), are recognizable. The highly hydrophilic nature of residue R in model GARHypG may contribute to such deviation *via* strong residue-water interactions. The domain 18 models exhibit distinguishable differences in representative structural ensembles, underlining the high flexibility of this disordered region, given its discernible conformational heterogeneity qualitatively. We find variability in structural ensembles based on cluster distributions identified through Jarvis Patrick clustering, namely, 533 clusters for the Non-Hyp model, 586 clusters for the D18-1 model, and 523 clusters for the D18-2 model. The full-length tropoelastin models likewise reveal remarkable variations in their representative structural ensembles, some of which adopt an elongated structure with a longer end-to-end length, such as model 5hyp-2, 8hyp-2, 9hyp-1, and 17hyp-1, while some favor a wider molecular shape with a shorter end-to-end length, as model 17hyp-2.

The prevalent variations observed in representative ensembles, obtained from Jarvis Patrick clustering, across the three different sequence length scale models evidently imply that the introduction of prolyl hydroxylation perturbs protein dynamics. Owing to the high number of degrees of freedom in highly disordered molecules such as tropoelastin, Hyp-containing models at scales beyond short peptides undergo substantial conformational changes. Consequently, these models display distinct structural ensembles at equilibrium, with localized alterations propagating widely across the entire molecule.

### Prolyl hydroxylation enhances local protein-solvent interactions *via* hydrogen bonding

**Figure 4:**
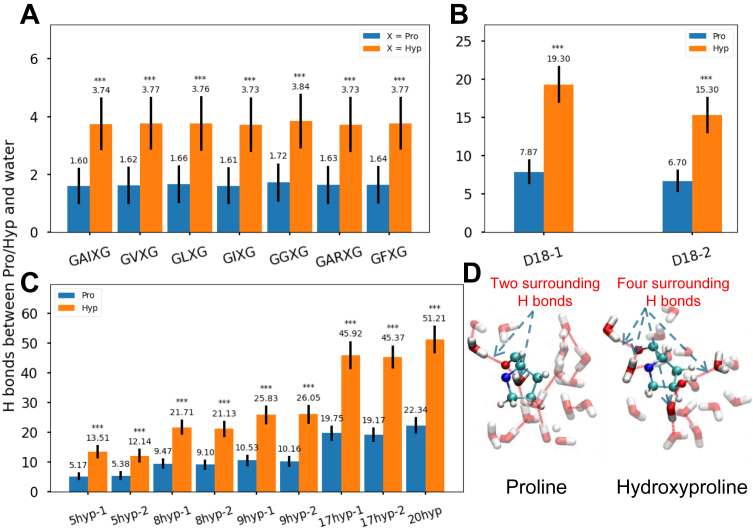
**Hydrogen bonding patterns across elastin models of different lengths.** Local hydrogen bonding patterns between proline versus hydroxyproline and water (*A*) in the motif models, (*B*) in the domain 18 models, (*C*) in the full-length tropoelastin models, and (*D*) presented in cartoon representations. Degrees of significance are indicated in the figure captions as follows, ∗*p* < 0.05, ∗∗*p* < 0.01, ∗∗∗*p* < 0.001.

We compare the number of hydrogen bonds formed between proline and hydroxyproline residues and their surrounding water molecules. Our results indicate that hydroxyproline residues have a greater propensity to form hydrogen bonds with water than proline residues (Fig. 4, *A*–*C*). In the motif models, hydroxyproline forms two more hydrogen bonds with water on average compared to proline (Fig. 4*A*). Analogously, hydroxyproline residues in the domain 18 models (Fig. 4*B*) and the full-length tropoelastin models (Fig. 4*C*) form twice as many hydrogen bonds with water as proline residues do. Figure 4*D* shows representative snapshots of the hydrogen bond network within a cutoff distance of 5 Å of Pro *versus* Hyp, substantiating the mechanism for the stronger association between Hyp and water.

**Figure 5:**
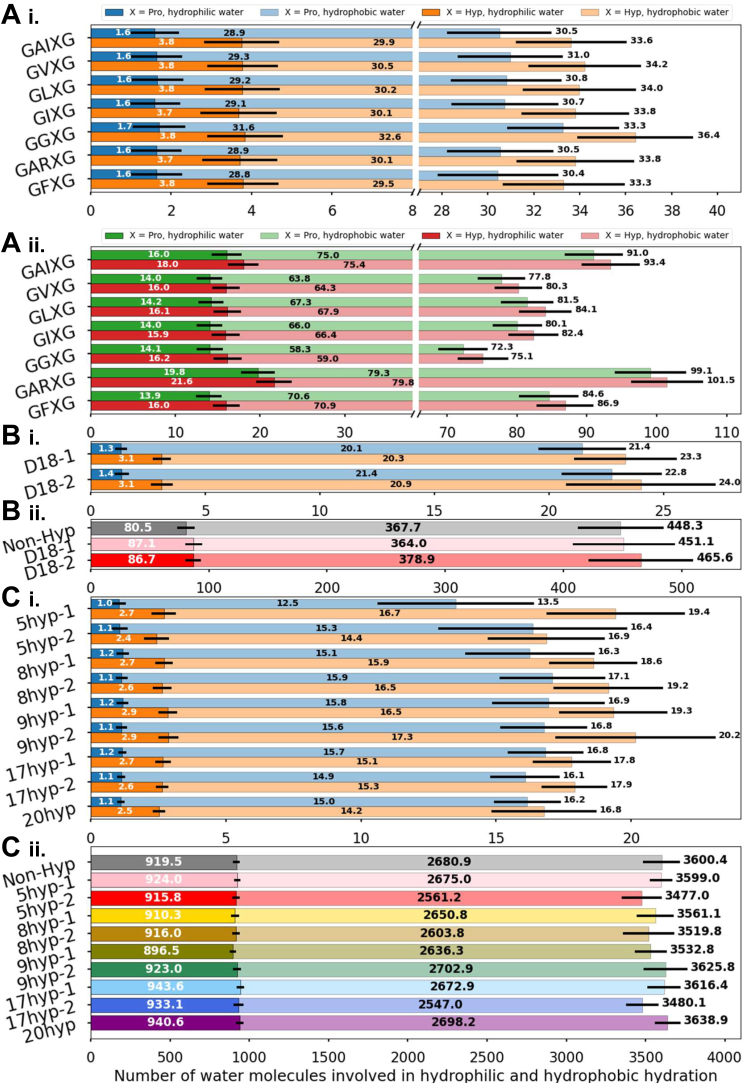
**The distributions of local hydration water and global hydration water.** (*A*, i) number of all hydration water molecules and hydrophilic hydration water molecules around each proline versus hydroxyproline in the motif models. (ii) number of all hydration water molecules and hydrophilic hydration water molecules around nonhydroxylation *versus* hydroxyproline-modified motif models. (*B*, i) number of all hydration water molecules and hydrophilic hydration water molecules around each proline *versus* hydroxyproline on average in the domain 18 models. (ii) number of all hydration water molecules and hydrophilic hydration water molecules around nonhydroxylation versus hydroxyproline-modified domain 18 models. (*C*, i) number of all hydration water molecules and hydrophilic hydration water molecules around each proline *versus* hydroxyproline on average in the full-length tropoelastin models. (ii) number of all hydration water molecules and hydrophilic hydration water molecules around nonhydroxylation *versus* hydroxyproline-modified the full-length tropoelastin models.

We also evaluate the water distribution locally (local refers to around the modification site; in this case, Pro *versus* Hyp), and globally (global refers to around the entire molecule; in this case, Non-Hyp *versus* Hyp-containing molecule). Figure S2 reveals the local water density around the modification site across different sequence lengths, where we find a sharper peak in the first hydration layer (∼2 Å) of Hyp compared to that of Pro, corresponding to enhanced hydrophilic hydration. This finding agrees with the increased residue-water hydrogen bonding surrounding Hyp shown in Figure 4. In addition, we analyze the quantity of nearest hydration water at both the local and global level (Fig. 5, *A*–*C*). Locally, we observe more hydration waters surrounding each Hyp residue compared to each Pro residue in the motif models (Fig. 5A (i)). This finding can be attributed to a higher proportion of hydrophilic hydration in relation to total hydration for Hyp as compared to Pro (Fig. 5A (i)). We observe similar trends in the domain 18 models (Fig. 5B (i)) and the full-length tropoelastin models (Fig. 5C (i)). Globally, we also find more hydration waters surrounding the entire Hyp-containing motif models compared to unmodified motif models (Fig. 5A (ii)), corresponding to a higher proportion of hydrophilic hydration in relation to total hydration for Hyp-containing motif models. We observe similar global hydration water distribution patterns in the domain 18 models (Fig. 5B (ii)). However, for the full-length tropoelastin models, we do not find an increase in either hydrophilic or total hydration water surrounding the hydroxylated full-length tropoelastin compared to the unmodified version (Fig. 5C (ii)). In addition, no correlation is observed between the number of hydroxylated prolines (Hyps) and hydration water content in the full-length tropoelastin models. We conclude that in motif and domain 18 models, prolyl hydroxylation not only increases the total number of local hydration water molecules through enhanced hydrophilic interactions, which corroborates the aforementioned hydrogen bonding pattern, but also consistently strengthens the global hydration effects. The full-length tropoelastin models, however, reveal intriguing disparities between local and global hydration patterns. For each substitution site, Hyp still manifests its affinity to water, as evidenced by a rise in hydrophilic hydration in relation to total hydration, compared to Pro. However, the hydrophobic and hydrophilic regions of Hyp-modified tropoelastin may further undergo extensive structural rearrangement, consistent with tropoelastin’s flexible nature. This rearrangement results in reduction in surface hydrophobic hydration water, leading to a decrease in total hydration water content, as seen in 5hyp-1, 5hyp-2. 8hyp-1, 8hyp-2, 9hyp-1, and 17hyp-2. We note that the hydration layer of the protein is highly fluctuating based on the large error bars in Figure 5C (ii), confirming the dynamic nature of the water–protein interactions in this system. Consequently, Hyp-containing tropoelastin models may not consistently demonstrate an increase in global hydration water counts.

**Figure 6:**
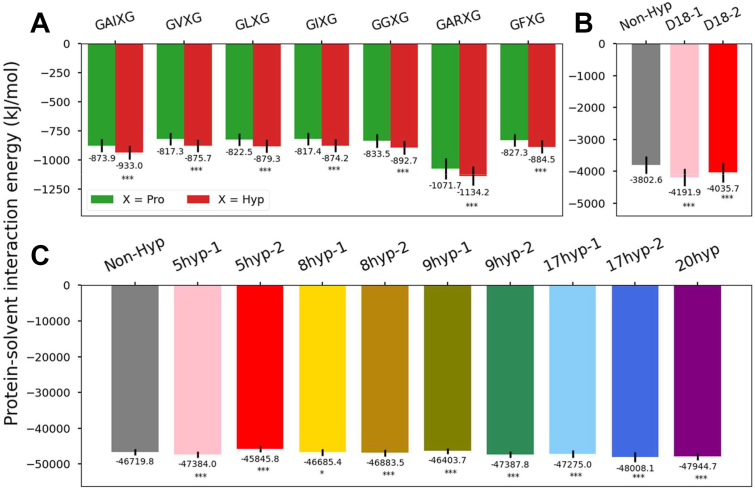
**The interaction energy between protein and solvent in elastin models**. Protein-solvent interaction energy comparison among (*A*) the motif models, (*B*) the domain 18 models, and (*C*) the full-length tropoelastin models. Degrees of significance are indicated in the figure captions as follows, ∗*p* < 0.05, ∗∗*p* < 0.01, ∗∗∗*p* < 0.001.

Figure 6 shows the global protein-solvent interaction energy for three models of different scales. Interestingly, both Hyp-containing motif models and domain 18 models exhibit higher affinities toward water (Fig. 5, *A*–*C*), implying that these Hyp-containing models may require higher energy to disrupt their hydration layers, potentially indicating increased structural rigidity and stability. However, this trend is not universally observed across all Hyp-containing full-length tropoelastin models, especially in model 5hyp-2, 8hyp-1, and 9hyp-1. The comparatively less pronounced results at the larger system size may be attributed to prolyl hydroxylation-induced changes in the energy landscape. It appears that tropoelastin, in order to minimize energy, undergoes structural rearrangement that modifies the hydration layer.

### Stronger interactions with water by hydroxyproline promote local rigidity characterized by reduced accessible conformations and reduced local fluctuation

**Figure 7:**
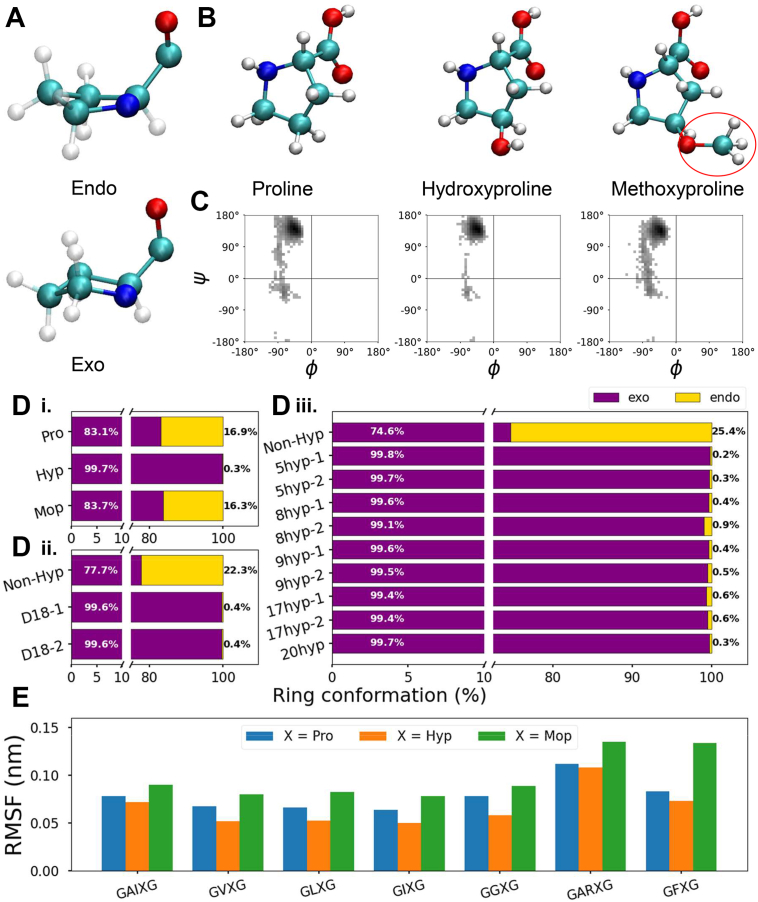
**Local conformations of proline versus hydroxyproline***A*, common ring conformation adopted by proline residues. *B*, cartoon representations of proline, hydroxyproline, and methoxyproline (structural difference as shown in the *red circle*). *C*, Ramachandran plots of proline *versus* hydroxyproline *versus* methoxyproline in the motif model, GAIXG, with X as Pro, Hyp, or Mop, respectively, showing the mainchain conformations. *D*, the preferred side chain conformations (%) of (i) proline *versus* hydroxyproline *versus* methoxyproline in the motif model, (ii) proline *versus* hydroxyproline in the domain 18 model, (iii) proline *versus* hydroxyproline in the full-length tropoelastin model. Degrees of significance are indicated in the figure captions as follows, ∗*p* < 0.05, ∗∗*p* < 0.01, ∗∗∗*p* < 0.001. *E*, root mean square fluctuation (RMSF) of proline *versus* hydroxyproline *versus* methoxyproline in the motif models. Mop, methoxyproline.

The pyrrolidine ring of proline and its derivatives may adopt two conformations, endo or exo (Fig. 7*A*), depending on the side chain conformation, specifically dihedral angle χ1, defined by atoms N, C-α, C-β, and C-γ and dihedral angle χ2, defined by atoms C-α, C-β, C-γ, and C- δ (18). The endo conformation is defined by χ1 > 0 and χ2 < 0 and the exo conformation is defined by χ1 < 0 and χ2 > 0 ^18^. Studies on hydroxylation of collagen suggest that the exo conformation of proline and its derivatives can be stabilized by the *gauche* effect, which is characterized by the interaction between the backbone nitrogen (N_i_) and the hydroxyl oxygen (O^δ1^_i_) in the current residue (illustrated in Fig. S3). Another important conformational heterogeneity of proline and its derivatives is associated with cis/trans isomerism, determined by dihedral angle ω (0° for cis and ±180° for trans) (19). The dihedral angle ω is defined by four atoms: C-α and C from the last neighboring residue, and N and C-α from the current residue (illustrated in Fig. S3) (19). Studies on hydroxylation of collagen found that the preference of adopting the trans isomer by proline and its derivatives can be attributed to the n → π∗ interaction. The n → π∗ interaction is characterized by the delocalization of the lone pair of electrons on the backbone carbonyl oxygen of the last neighboring residue (O_i-1_) into the backbone carbonyl group of the current residue (− C_i_ = O_i_ −) (20, 21, 22). Moreover, the *gauche* effect-stabilized exo conformation in collagen can further enhance the n → π∗ interaction by generating a favorable distance and angle for the electron delocalization from O_i-1_ into – C_i_ = O_i_ −, resulting in the preference of the trans isomer (23). Therefore, these two stereoelectronic effects, namely, the *gauche* effect and the n → π∗ interaction, present in 4I-prolyl hydroxylated collagen, can preorganize torsion angles, resulting in the exo ring pucker and the trans isomer, which thereby impart structural stability to the collagen triple helix (20, 21, 23, 24, 25).

In contrast with findings of prolyl hydroxylation of collagen, we postulate that the enhanced hydrophilic hydration of Hyp of tropoelastin gives rise to fewer accessible conformations and reduced fluctuations locally (at the hydroxylated sites), which confers structural rigidity and thereby possibly further alters the properties of elastin, such as interactions with other molecules or cellular components. To substantiate our hypothesis about the potential mechanism of rigidity at the hydroxylated sites in elastin, we consider another proline substitution, methoxyproline (Mop), where the electron-withdrawing hydroxyl oxygen and all the associated stereoelectronic effects are retained, but hydrophilic hydration is reduced *via* the substitution of a methyl group for the hydroxyl hydrogen (Fig. 7*B*) (20, 21, 24, 26), as a positive control in our investigation on motif models.

The local conformations of Pro, Hyp, and Mop demonstrate different distribution patterns (Fig. 7, *C* and *D*, Figs.S5 and S6). The distribution of accessible Φ, Ψ dihedral angles in Hyp in the motif models is pronouncedly narrower than Pro and Mop, suggesting reduced accessible conformational space (more conformational stability) of the main chain in Hyp (Figs. 7*C*, S4, *A*–*C*). By contrast, the distribution of accessible main chain Φ and Ψ dihedral angles of Mop in the motif models is wider compared to Pro, indicating increased accessible conformational space (less conformational stability) of the main chain in Mop (Figs. 7*C*, S4, *A*–*C*). In addition, while Pro and Mop only exhibit the exo conformation for 80% ∼ 85% of the simulation time in the motif models, Hyp adopts the exo conformation prevalently, for more than 99% of the simulation time (Figs. 7D (i), S5). Despite retaining the same *gauche* effect, as present in Hyp, we do not find a similar distinct preference of Mop for the exo ring pucker compared to Hyp. Also, when compared to Pro where the *gauche* effect is absent, Mop does not show a more stabilized exo conformation. These findings imply that there is little association between the *gauche* effect and the preference for the exo conformation in tropoelastin. Moreover, the stronger preference for the exo conformation of Hyp than that of Pro as evident in the motif models remains consistent in the domain 18 models and the full-length tropoelastin models (Fig. 7*D* (ii-iii)). This confirms the correlation of conformational analysis at different sequence lengths. In sum, the favored exo conformation of Hyp in tropoelastin is less likely to be associated with the *gauche* effect. Instead, we speculate that the enhanced hydrophilic hydration of Hyp, as determined in Implications of prolyl hydroxylation for coacervation, cross-linking, functional needs, disease, and aging, contributes to the conformational stability of both the main chain and the side chain.

Because studies in collagen have shown that the exo conformation can reinforce the trans isomer, we also calculate dihedral angle ω to determine which isomer (cis/trans) is adopted by Pro, Hyp, and Mop in elastin, and whether the preference for adopting the trans isomer is associated with a higher tendency of adopting the exo conformation. As shown in Figure S6, Pro, Hyp, and Mop always adopt the trans isomer in the motif models, and we determine no distinct difference in the preferred isomer among the three residues. Despite a greater propensity for adopting the exo conformation, which can potentially enhance the n → π∗ interaction in favor of the trans isomer, we do not find a stronger preference of Hyp for the trans isomer. Also, because Pro, Hyp, and Mop always adopt the trans isomer, we cannot determine if and how much the n → π∗ interaction influences such propensity. These findings do not suggest a strong association between the preferred exo and trans conformations within tropoelastin and the stereoelectronic effects, namely, the *gauche* effect and the n → π∗ interaction.

We further examine the root mean square fluctuation (RMSF) of Pro versus Hyp versus Mop, as another metric of rigidity and stability. As expected, despite the plausible speculation of stereoelectronic effects-driven rigidity, the local fluctuation of Mop does not reflect a reduction in the RMSF values, compared to that of Pro. In fact, the local fluctuation even increases in some models (Fig. 7*E*), such as GFMopG. By contrast, the local fluctuation of Hyp reveals consistent decrease across the motif models (Fig. 7*E*), the domain 18 models and the full-length tropoelastin models (Fig. S7), compared to Pro. Notably, we observe that Mop in motif models forms one additional hydrogen bond compared to Pro, and one fewer hydrogen bond compared to Hyp (Fig. S8). Such difference between Mop and Hyp in hydrogen bonding can be ascribed to the substitution of a methyl group for the hydroxyl hydrogen, and thereby, reduced hydrophilic hydration limited further enhancement in local rigidity. Together with the reduction in the RMSF values of Hyp and increase in those of Mop, these findings suggest that the local rigidity in hydroxylated elastin is driven by enhanced hydrophilic hydration. This contrasts with the mechanism associated with collagen triple helix stabilization, which stems from stereoelectronic effects.

Therefore, based on the analysis presented in Implications of prolyl hydroxylation for coacervation, cross-linking, functional needs, disease, and aging and The stabilizing role of prolyl hydroxylation in elastin versus collagen, we conclude that in tropoelastin, it is mainly the stronger hydrophilic hydration featured in hydroxyproline (*i.e.*, enhanced hydrogen bonding around the hydroxyl group, as shown in Fig. 4), rather than the stereoelectronic effects involving its hydroxyl oxygen and the carbonyl group, that engender local rigidity characterized by a reduced number of main chain conformational states and side chain conformational states, as well as reduced local fluctuations.

### Prolyl hydroxylation may reduce degradation susceptibility and cross-linking efficiency of elastin

Apart from hydration-induced decrease of local fluctuations at the Hyp positions, the subsequent impacts of hydration on overall fluctuations at the global scale of the molecule are also noteworthy (Fig. S7). Across all models of the Hyp-modified full-length tropoelastin, the root mean square fluctuations per residue are considerably lower than those in the Non-Hyp model, suggesting impaired local flexibility and altered overall protein dynamics due to enhanced hydration effects *via* the introduction of Hyp. This observation agrees with observations in the literature that hydration water strongly correlates with protein dynamics (27, 28). Despite conflicting opinions on whether the dynamics of hydration water is faster (29) or slower (30) around intrinsically disorder proteins (IDPs), previous studies have suggested that protein and water mutually affect and shape each other, exhibiting a coupling motion (6, 27). We postulate that the enhanced local hydration effects around elastin may induce slower water dynamics, thereby restricting protein flexibility, and as a result yielding lower per residue fluctuation (31).

**Figure 8:**
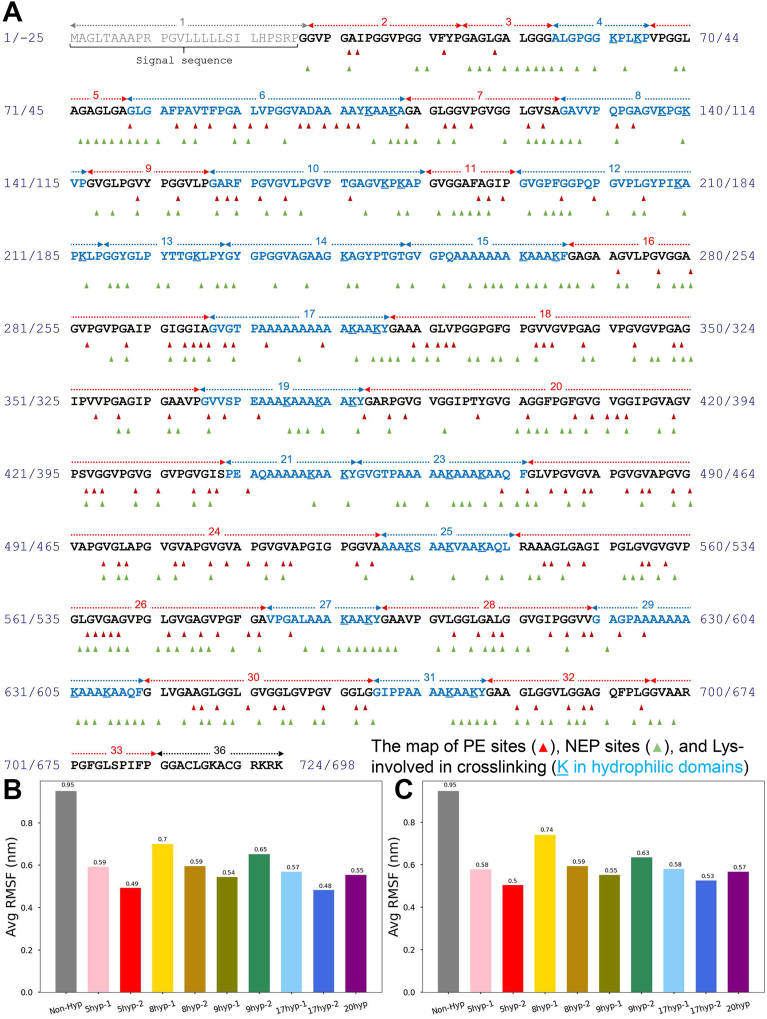
**Analysis of fluctuations at elastase-targeting sites in full-length tropoelastin models**. *A*, the tropoelastin sequence map showing PE targeting sites marked in *red triangles*, NEP targeting sites marked in *green triangles* and hydrophilic domains colored in *blue* with crosslinking-involved lysine residues underlined. *B*, the average RMSF values of the PE targeting sites. *C*, the average RMSF values of the NEP targeting sites. PE, pancreatic elastase. NEP, neprilysin; RMSF, root mean square fluctuation; PE, pancreatic elastase.

**Figure 9:**
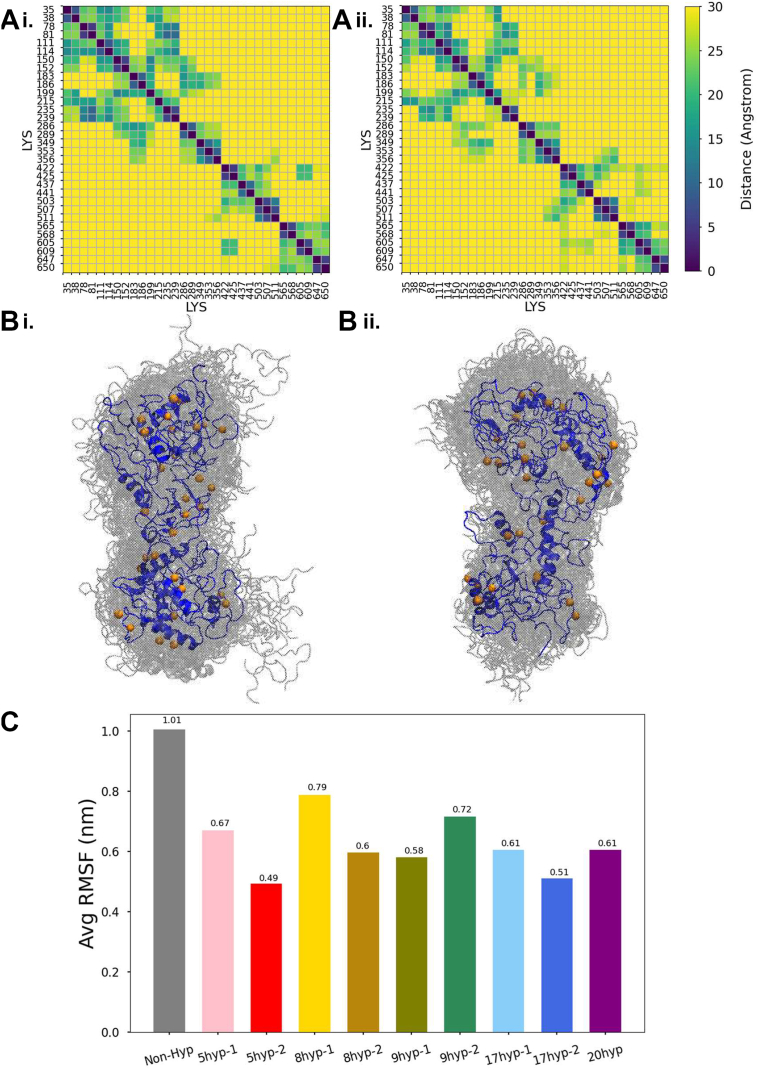
**Prediction of cross-linking efficiency in full-length tropoelastin models.***A*, comparison of lysine-lysine distance contact map between (i) the nonhydroxylated full-length tropoelastin models and (ii) the 20hyp full-length tropoelastin model (overhydroxylation). *B*, cartoon representation of lysine-lysine pairs in (i) the nonhydroxylated full-length tropoelastin ensemble and (ii) the 20hyp full-length tropoelastin ensemble (overhydroxylation). Each lysine residue is annotated by *orange beads*. *C*, the average RMSF values of lysine residues in the hydrophilic domains. RMSF, root mean square fluctuation.

Further, protein dynamics play an essential role in maintaining the protein’s activity (32, 33), which not only consequently impacts protein functions, including protein folding (34), protein assembly (34), catalysis (35, 36), and cross-membrane transportation, (34, 36), but also may indirectly affect processes like transcription and translation (34, 37), by impacting the activity of other proteins involved in those mechanisms. As the local flexibility in Hyp-modified tropoelastin is reduced, the ability to move between different conformational states, which is critical for some protein functionalities, may also be impaired. In particular, the finding of lower fluctuation in Hyp-modified models may explain reduced enzymatic degradation of tropoelastin mediated by proteases, pancreatic elastase and neprilysin (NEP) (38, 39). Elastase-targeting sites, subject to pancreatic elastase and NEP, have been identified by MS (Fig. 8*A*) and display a reduction in averaged RMSF in the presence of hydroxyproline in the models of full-length tropoelastin (Fig. 8, *B* and *C*). We infer that the enzymatic susceptibility toward degradation of tropoelastin is impeded as a consequence of compromised protein dynamics. Likewise, the secondary structures of PE-/NEP- targeting sites exhibit discernable differences across different systems of the full-length tropoelastin models (Fig. 9*A* (i-ii)). Such difference implies structural heterogeneity and possible alterations in the distribution of these involved sites on the protein’s surface, which may alter the propensity of proteases to recognize targeting sites.

Lysine residues residing in hydrophilic domains are involved in enzymatic cross-linking, which is essential for the formation of mature elastic fibers (relevant lysine positions are annotated in Fig. 8*A*). Figure 9*A* describes the lysine-lysine contact map within the Non-Hyp and the 20hyp (overhydroxylated) full-length tropoelastin models, showing a decrease in the proximity among lysine pairs in the overhydroxylated model compared to the nonhydroxylated model. The lysine positions in these two models are visualized in cartoon representations in Figure 9*B*. The spatial proximity between lysine pairs can provide implications for cross-linking possibilities: the closer the lysine pairs are, the higher chance that the cross-linking reaction takes place between the lysine pairs. Apparently, overhydroxylation can potentially reduce cross-linking possibilities within tropoelastin. To assess the spatial proximity of lysine pairs of the other Hyp-modified full-length models with those of the nonhydroxylated full-length model, we consider a series of cutoff distances, from 16 Å to 30 Å, within which lysine pairs are likely to form a cross-link (40). Interestingly, at a cutoff distance of 16 Å, only two out of nine models show a statistically significant decrease in the proximity among lysine pairs compared to Non-Hyp models. However, the number of models exhibiting a statistically significant decrease gradually rises with the cutoff distance up to a cutoff distance of 26 Å. At a cutoff distance of 26 Å, six out of nine models show a statistically significant decrease (Table S2). Notably, three models, 5hyp-1, 8hyp-1, and 17hyp-2, begin to reveal a statistically significant decrease in the proximity among lysine pairs compared to Non-Hyp models when the cutoff is set as 22 Å, 20 Å, and 18 Å, respectively. Our results indicate that a number of lysine pairs in Non-Hyp models may shift positions from an interresidue distance of 20 Å ∼ 22 Å, to a larger distance, up to 30 Å, where cross-linking is less likely to occur. This effect is particularly noticeable in models 9hyp-2, 17hyp-2, and 20hyp. Such heterogeneity of lysine contacts in different full-length models again confirms extensive structural variability, and the resulting deviating patterns of cross-linking. In addition, we observe a decrease in averaged lysine RMSF (Fig. 9*C*), suggesting that lysine residues in Hyp-modified tropoelastin are less mobile. Given the importance of residue flexibility necessary for protein activities, *e.g.*, the recognition of lysine by enzymes involved in cross-linking processes, we postulate that the reduced lysine fluctuation would potentially decrease cross-linking efficiency in Hyp-modified tropoelastin.

## Discussion

### Hydration effects vary at different length scales in flexible elastin

In this study, we find divergent results concerning local (Fig. 5*C*) and global hydration effects (Fig. S10) across models of different sequence length. Hydration effects are characterized by the number of hydrogen bonds and hydration water molecules. Locally we see an increase in both hydrogen bond number and hydration water number; however, this trend is not consistently reflected globally. Specifically, we do not find an enhancement in the number of hydrogen bonds and hydration water molecules in 7 of 9 full-length Hyp-modified tropoelastin models, despite the presence of enhanced local hydration effects. Previous studies on IDPs have also noted the potential inconsistency of properties between IDPs with shorter sequences and those with longer sequences (41, 42, 43). The extent of disorder in IDPs with a longer length is typically more difficult to predict (43, 44). Given the sizeable presence of disordered regions within tropoelastin, which engenders extensive dynamic conformational reorganization, domains may undergo structural rearrangements and then closely associate with each other. Therefore, the measurements of global hydration effects for a structural ensemble, specifically, hydrogen bond number, hydration water number, and protein–water interaction energy, may not necessarily show a linear trend directly correlated with local hydration effects. Consequently, local enhanced hydration effects may lead to nonlinear downstream changes in hydration behavior of elastin at higher scales, *e.g.*, in elastin aggregates derived from various species, where water content is reported to be between 40% and 60% (45); and in elastin fibers, which are reported to contain 250 to 300% of water in porcine aorta (g water per 100 g elastin) (46, 47). The hydration water of elastin fibers includes both intrafibrillar and extrafibrillar water, and the intrafibrillar water establishes direct interactions with elastin molecules (47, 48). Therefore, alterations in elastin hydration effects at a molecular scale can have downstream impacts on hydration effects at a fiber scale.

### Implications of prolyl hydroxylation for coacervation, cross-linking, functional needs, disease, and aging

#### Implications of prolyl hydroxylation in coacervation

Structural rearrangement induced by prolyl hydroxylation results in altered intraprotein contacts (Fig. 2*C*), as well as a rise in energy needed for inverse temperature phase transition (10, 49). This, in turn, can impact coacervation, multimer aggregation, and cross-linking behavior, ultimately leading to the formation of distinct higher-order structures. Prolyl hydroxylation increases the transition temperature for coacervation, and therefore favorable conditions, such as a higher concentration or the presence of a coacervation-promoting solvent, *e.g.* trifluoroethanol (TFE), are required to overcome the higher energy barrier for coacervation to occur for Hyp-containing ELPs, compared to nonhydroxylated ELPs. In addition, Hyp-containing ELPs tend to form globular aggregates instead of fibers (8, 10, 50). Once suspecting that such distinct patterns in coacervation and resultant elastin aggregates could result from the unstable secondary structures due to the presence of Hyp (10, 51), Urry *et al.* later attributed this phenomenon to the enhanced hydrophilicity (or, weakened hydrophobicity) introduced by the presence of Hyp, which impedes hydrophobic regions from associating and then collapsing (49, 52). Bochicchio *et al.* proposed a similar mechanism based on their experimental study using ELPs (8). Our simulation results suggest that prolyl hydroxylation indeed induces structural rigidity and reduces protein dynamics through hydration effects. The strengthened hydration layer, especially at a local level, shields the hydrophobic domains from collapsing. As a result, the energy barrier of tropoelastin molecules to coacervate increases. This finding suggests that targeted hydroxylation can be employed to tune the phase transition temperature of elastin-based materials. While our models suggest a molecular mechanism that accounts for changes in coacervation behavior between modified and unmodified elastin, future work could directly explore these effects using large-scale models, such as multimer aggregate elastin models.

#### Implications of prolyl hydroxylation in cross-linking

Our study reveals that prolyl hydroxylation reduces lysine contacts in tropoelastin, suggesting a decreased propensity for intraprotein cross-link formation among these lysine residues within the monomer. This finding is consistent with previous experimental results where hydroxylated elastin molecules, induced by incubation with ascorbate, undergo reduced cross-link formation but enhanced solubility in water (52). This phenomenon may be attributed to the increased local hydration of elastin in the presence of hydroxyproline, leading to extensive conformational changes and subsequently altering the spatial distribution among lysine pairs.

#### Implications of prolyl hydroxylation in fulfilling functional needs of elastin and its role in elastin-related diseases

We note that in healthy organisms, variability in prolyl hydroxylation may serve functional needs within specific organs and tissues in the body. For instance, hydroxylation degree of specific proline residues is found to be higher in IVD as compared to other tissues; since IVD is subject to high compressive loads, prolyl hydroxylation may play a role in supporting the compressive strength of the IVD (7).

Overhydroxylation of prolyl residues, however, may have implications in elastin-related disease. The expected inhibition of coacervation due to prolyl hydroxylation suggests that very few “properly” associated tropoelastin molecules in the overhydroxylation case would undergo the canonical subsequent steps in elastogenesis, including multimer aggregation, intramolecular and intermolecular cross-linking, and deposition on to a microfibrillar scaffold. As manifested in Bochicchio’s experimental study, ELPs of an elastin representative sequence (VGVPG)_10_, with an extreme case where hydroxylation of proline occurs in all 10 monomeric units, exhibited densely packed globular aggregates *in vitro*, in contrast to the fiber formation observed for nonmodified ELP (8). As this motif commonly occurs in the sequence of full-length tropoelastin molecules, it is plausible that the assembly of tropoelastin with excessive prolyl hydroxylation also deviates from canonical fiber-like morphology, thereby potentially negatively influencing the functionality of elastin, leading to elastin-related diseases.

The potential deficiency of elastin cross-linking due to overhydroxylation may also have implications in certain elastin-associated diseases. As one example, increased hydroxyproline content in elastin of aortic connective tissues was found in patients with Marfan syndrome (53). In addition, reduced elastin cross-linking was detected. Our findings suggest that distances between lysine pairs may be increased in Hyp-modified elastin, which may provide a mechanistic explanation for the observed differences. Furthermore, hydroxyproline is associated with altered morphology of elastin aggregates, which may also have negative implications for elastin functionalities (8). We infer that in these patients insufficient cross-linking of elastin may likely be driven by prolyl hydroxylation-induced changes, playing an essential role in the etiology of Marfan syndrome. Other studies identify insufficient cross-linking of elastin with a myriad of complications, including thoracic aortic aneurysms (54, 55), dissections (54, 55), and improper alveolarization during lung development (56). Further investigation into the possible associated presence of modified hydroxylation activity may be interesting to probe as a possible contributing mechanism in these conditions.

#### Implications of prolyl hydroxylation in aging

The loss of hydration water around elastin alters its mechanical properties, resulting in increased brittleness and rigidity (47). Weaker mechanical properties may contribute to mechanical failure, a hallmark of aging elastin fibers (57). With the presence of prolyl hydroxylation, however, elastin is capable of retaining more surrounding hydration water, at least at a local scale. The hydration water can help hydrate elastin *via* a stronger hydration shield, mitigating stiffening and loss of elasticity induced by water loss, and thereby preserving its mechanical functions. We infer that hydroxylation may serve as an evolutionary mechanism to protect the long-lived elastin protein and elastic tissues from aging-associated degeneration by maintaining the hydration required for optimal molecular and tissue function.

Another important hallmark of elastin aging is proteolytic damage by enzymatic cleavage (57). The presence of prolyl hydroxylation in elastin may leverage the locally enhanced hydration effects to reduce the mobility of elastase-targeting sites, consequently decreasing the occurrences of enzymatic cleavage events. Our findings align with those of Bochicchio’s experimental study, where fewer individual peptides are found to be hydrolyzed from a Hyp-modified ELP compared to an unmodified ELP (8). As a result of potential changes to enzymatic degradation, Hyp-modified elastin may be subject to a different half-life in the organism. Based on these aspects, prolyl hydroxylation of elastin may have a mitigating impact on age-associated degeneration of the elastic fiber, and contribute to a longer biomechanically functional lifetime.

### The stabilizing role of prolyl hydroxylation in elastin *versus* collagen

Our simulation results support enhanced hydrophilic hydration in Hyp as the mechanism through which prolyl hydroxylation gives rise to structural rigidity in elastin. In Hyp-modified elastin models, we find decreased main chain accessible conformational states of Hyp, a stronger tendency to adopt the exo conformation (*i.e.*, decreased side chain accessible conformational states), and reduced protein mobility as reflected in decreased RMSF values at local (Hyp) and global (the full-length tropoelastin) scale, compared to Pro in nonhydroxylation models. These data suggest structural rigidity results from prolyl hydroxylation, characterized by stabilized conformations and reduced protein fluctuations. We then examined Mop, where the stereoelectronic effects are similar to Hyp but its propensity for hydrophilic hydration, *i.e.*, the ability to form hydrogen bonds with water, is lower than Hyp. These stereoelectronic effects include the *gauche* effect and the n → π∗ interaction, where the *gauche* effect is exerted by the substitution of hydroxyl oxygen that strengthens the exo conformation. The exo conformation further stabilizes the n → π∗ interaction, resulting in the preference of adopting the trans isomer (20, 21, 23, 24, 26, 58). Due to the hydroxyl oxygen substituent present in both Hyp and Mop, the resultant *gauche* effect may be expected to also theoretically stabilize the exo conformation in Mop. However, in Mop-modified elastin motif models, we identify increased main chain accessible conformational states of Mop, a slightly lower tendency to adopt the exo conformation (*i.e.*, increased side chain accessible conformational states), and higher mobility of Mop as shown by its greater RMSF values, compared to Hyp. We also find that the tendency of adopting the exo conformation and the mobility of Mop is comparable to that of Pro, suggesting little influence from the *gauche* effect on the structural rigidity. In addition, since the *gauche* effect-stabilized exo conformation can theoretically enhance the n → π∗ interaction, leading to the trans isomer, the stronger preference for the exo conformation of Hyp compared to Mop and Pro may be expected to give rise to a higher tendency toward the trans isomer of Hyp. Nevertheless, we find Hyp, Mop, and Pro always adopt the trans isomer during production runs and cannot determine if and how much the n → π∗ interaction is involved in such preference. These results contradict the possibility that prolyl hydroxylation influences elastin *via* stereoelectronic effects. Also, our study partially corroborates an experimental study of an ELP, sequence VGVXGVG, where X = Pro, Hyp, and Mop (59). The authors find more folded secondary structures for Mop in this ELP compared to Hyp. This result supports how reduced hydration, induced by the 4(R) methoxyl group in Mop, can influence the peptide conformation, which further enhances the self-assembling propensities of Mop-containing ELP, compared to Hyp-containing ELP. This study also measures Kt/c, the ratio between the trans state *versus* the cis state adopted by Pro/Hyp/Mop-included ELPs, in order to determine their preferences for the isomers. Interestingly, both Hyp- and Mop-containing ELPs show much higher Kt/c values than Pro-containing ELPs, suggesting stronger propensities of Hyp- and Mop-containing ELPs to adopt the trans isomer, compared to the Pro-containing ELP. This discrepancy between their results and our simulation results can be explained by the time scale limit of MD simulations. Still, these experimental results do not fully support the contribution from stereoelectronic effects to structural rigidity in Hyp-modified elastin; instead, we believe increased hydrophilic hydration of Hyp is the driving mechanism for the enhancement of structural rigidity of Hyp-modified elastin.

Prolyl hydroxylation is also common in other proteins, such as collagen, another prevalent protein in connective tissues and organs that provides essential mechanical support. Hyp in collagen often appears within a triple amino acid pattern, Pro-Hyp-Gly, making up 10.5% of all triplets composing the molecule (23, 60). In contrast to the hydration effects by which prolyl hydroxylation realizes its function in elastin, prolyl hydroxylation in collagen relies on stereoelectronic effects in order to enhance the structural stability of the collagen triple helix and increase thermal resistance of collagen.

Prolyl hydroxylation in collagen relies on the aforementioned stereoelectronic effects to fulfill its functions. Unlike tropoelastin, Hyp and Mop in collagen have a higher tendency to adopt the exo conformation, while Pro commonly adopts the endo conformation (23, 24). This is consistent with the observation that the *gauche* effect can enhance the exo conformation of the ring pucker of Pro and its derivatives in collagen. Also, Mop and Hyp in collagen-like polymers show a strong preference for the trans isomer compared to Pro, which confirms the expected contribution from the *gauche* effect that further strengthens the n → π∗ interaction (21, 23).

Hydroxylation results in changes to mechanical properties in collagen: in comparison to unmodified collagen, Hyp-modified collagen exhibits lower stiffness and lower Young's modulus (61). In addition, the incorporation of Hyp also improves the folding rate of collagen, likely as a result of the stabilizing effects of hydroxyproline on the triple helix (62). For elastin, the influence of prolyl hydroxylation on mechanical properties at the nanoscale and folding has not been elucidated. Further research could delve into the molecular mechanisms underlying the mechanical disparities between hydroxylated and nonhydroxylated elastin. Although prolyl hydroxylation in elastin is partial and not a prerequisite for secretion as part of the elastic fiber assembly, further investigations are necessary to elucidate the exact functional roles of this PTM (63).

Overall, our study demonstrates that prolyl hydroxylation also modulates the functional properties of elastin, yet in a different fashion compared to collagen, and with unique outcomes. In elastin, prolyl hydroxylation alters protein behaviors and functionalities *via* enhanced local hydration effects, including regulating elastogenesis (increasing coacervation temperature and reducing cross-linking), fulfilling functional needs, contributing to disease-related consequences and possibly modulating aging-associated degeneration of the elastic fiber. Based on the potential modulating properties of prolyl hydroxylation, these insights can be used to develop and inform novel ELP-based materials tuned to desired functional needs and appropriate half-life.

## Conclusions and outlook

We perform classical atomistic MD simulations on elastin models of different length, originating from the tropoelastin monomer, namely, short elastin motifs, domain 18, and full-length tropoelastin, in the presence of various combinations of hydroxyproline in place of proline residues, to study the effects of hydroxylation in elastin. Our findings demonstrate that prolyl hydroxylation strengthens local hydration effects, particularly by enhancing protein–water hydrogen bonding. This gives rise to local rigidity, characterized by reduced accessible conformational states and fluctuations in the local environment of the elastin molecules. Consequently, the structural ensembles of elastin are altered, and the protein dynamics are dampened, potentially contributing to reduced enzymatic degradation and abnormal assembly and cross-linking behavior of elastin-like proteins, as observed in experimental studies. These findings, in conjunction with existing literature, underscore the critical role of prolyl hydroxylation in elastin function and its potential role in adapting to the varying mechanical needs of different connective tissues. In addition, they illuminate the process of native elastin assembly and offer valuable insights into the potential involvement of hydroxylation in the degeneration of elastin in aging and disease. Based on these findings, strategically placed hydroxyproline residues may offer a means of engineering elastin-based materials with desired properties. Nevertheless, further research is needed to elucidate the precise mechanisms by which hydroxylation regulates elastin properties, which could provide valuable insights for applications in tissue engineering and regenerative medicine.

## Experimental procedures

### Model systems

We propose three types of elastin-based models to study how prolyl hydroxylation impacts elastin function at different scales. We begin with short representative motifs where prolyl hydroxylation often occurs natively to study the fundamental mechanism of prolyl hydroxylation altering the protein’s activity at the scale of unit building blocks. We also consider domain 18 because of its intrinsic flexibility and the high occurrence of prolyl hydroxylation associated with this domain. We also study the full-length tropoelastin molecule as it is the fundamental monomeric building block of elastic fibers.

#### Motif models

We select representative sequences where prolyl hydroxylation specifically takes place in the full-length tropoelastin molecule based on MS data (7), namely, GAIPG, GVPG, GIPG, GLPG, GGPG, GARPG, and GFPG, as detailed in Figure 2*A*. The sequences after prolyl hydroxylation are GAIHypG, GVHypG, GIHypG, GLHypG, GGHypG, GARHypG, and GFHypG.

#### Domain 18 models

We build three models for domain 18 extracted from the sequence of native human elastin isoform 2 (IF 2): the nonhydroxylated model, GAAAGLVPGGPGFGPGVVGVPGAGVPGVGVPGAGIPVVPGAGIPGAAVP; the domain 18 model including 6 Hyp residues (D18–1) corresponding to a study by Bochicchio *et al*. (8), where not all the prolyl hydroxylation occurs within the representative motifs; the domain 18 model including 5 Hyp residues (D18–2) where prolyl hydroxylation occurs within the Gly-Yaa(-Zaa)-Pro-Gly motifs exclusively, consistent with the study by Schmelzer *et al.* (7). The latter two models are considered as overhydroxylation cases in contrast to native states. Former studies suggest that no more than 20% of Pro residues in the full-length tropoelastin sequence are modified, while 5 to 6 Pro were partially modified out of 9 Pro residues within domain 18 in the latter two models (7). The hydroxylation map of domain 18 models is summarized in Figure 2B (i).

#### The full-length tropoelastin models

We select the number of Hyp occurrences within the full-length tropoelastin models based on mass spectrometric data of four human elastin samples, extracted from skin and aorta, reported by Schmelzer *et al.* (Table S1) (55, 64), where the hydroxylation percentage ranges from 5.8% to 20% in these samples, corresponding to 5, 8, 9, and 17 Hyp occurrences out of a total of 86 Pro residues in native human elastin IF 2.

We employ the hydroxylation degree of a Pro site as one of the criteria to select hydroxylation positions. The aforementioned study by Schmelzer et al. identified the positions of in total 21 Pro residues that are partially hydroxylated (7). We note that as we include serine (Ser) in position 422 in place of glycine (Gly), consistent with our previous full-length tropoelastin model (65), Pro421 does not reside within a Gly-Yaa(-Zaa)-Pro-Gly motif, tagged for hydroxylation. Therefore, Pro421 is not considered to be hydroxylated in our IF 2 model. Among the other 20 hydroxylation sites, Pro190 and Pro360 are determined to be hydroxylated in over 70% of the elastin samples, so we keep these two positions always hydroxylated in the models. In addition, Pro615 is found 50% hydroxylated among all elastin samples, so half of our models exhibit this position in the unchanged state and half exhibit this position in the hydroxylated state. The hydroxylation degree of the remaining potential sites, however, remains unavailable from experimental studies. Therefore, we select the remaining hydroxylation positions randomly from the rest of the 17 confirmed sites using computer programs, Pro34, Pro116, Pro147, Pro283, Pro327, Pro337, Pro342, Pro347, Pro387, Pro405, Pro415, Pro427, Pro433, Pro551, Pro560, Pro578, and Pro658. The remaining hydroxylation positions are assumed to have the uniform probability to be chosen for models. We use PyMol to modify Pro residues into Hyp residues (66). We also include a nonhydroxylated native model without any prolyl hydroxylation and an overhydroxylated model that includes all potential hydroxylated sites. The details of Hyp modifications of the full-length tropoelastin models are described in Figure 3 and Table 1.

### MD simulation

#### Simulation setup

##### Simulation parameters

We use the Gromacs simulation package (67) version 2018 for our simulations, with the CHARMM36m protein parameter set for topology and force field parameters for elastin (68), and TIP3P for water molecules (69). We implement the particle mesh Ewald (PME) method to compute electrostatic forces with grid spacing of 1.6 Å (70). We set the cutoff radius as 12 Å for short-range electrostatic interactions and Lennard-Jones interactions.

We first restrain the bonding connecting hydrogen with protein backbone and apply the steepest descent algorithm to minimize the energy of the system for at most 50,000 steps. We then equilibrate the system for 100 ps in both NVT and NPT ensembles. For temperature equilibration, we adopt the V-rescale thermostat to couple protein and nonprotein groups to separate temperature baths of 300 K (27 °C), with 0.1 ps as the time constant (71). For pressure equilibration, we use the Parrinello-Rahman barostat for pressure coupling (72). We assign 2 ps as the time constant for isotropic pressure coupling, 1 bar as the reference pressure, and 4.5e^-5^ bar^-1^ as compressibility. Upon the end of equilibration stages, we remove position restraints and initiate 1000 ns production runs using the NPT ensemble. Across the stages of equilibration and MD production run, we harness the LINCS algorithm (73) to constrain covalent bonds with hydrogen atoms and the leap-frog algorithm (74) for integration every 1 fs during equilibration and every 2 fs during MD production runs.

##### Motif models

The motif models are spliced from the full-length tropoelastin model. Each model has two conditions, unmodified (Non-Hyp) and Hyp-modified, and each condition has three replicates. Each of these replicates undergoes 1000 ns MD simulations in a water box of 2.5 × 2.5 × 2.5 nm^3^, and results of analysis are averaged among the trajectories corresponding to production runs of these three replicates.

##### The domain 18 models

The domain 18 models are spliced from the full-length tropoelastin model. Each condition has 10 replicates, respectively. Each of these replicates undergoes 1000 ns MD simulations in a water box of 10 × 10 × 10 nm^3^, and results of analysis are averaged among the trajectories of these 10 replicates.

##### The full-length tropoelastin models

The starting structure of the full-length tropoelastin model is obtained from our previous study (65). Each condition has three replicates. Each of these replicates undergoes 1000 ns MD simulations in a water box of 15 × 15 × 15 nm^3^, and results of analysis are averaged among the trajectories of these three replicates. The convergence in each case was based on RMSD data in Figure S11.

#### Simulation analysis

We post process all simulation trajectories using Gromacs (67) and visualize them in visual molecular dynamics (VMD) (75). Each trajectory is 1000 ns long, and we retain 2500 frames for each trajectory. Each frame can be considered as a data point of a certain property. All measurements are performed as an average over the last 500 ns (1250 frames) of the production runs from all replicates. We perform clustering on the concatenated trajectories of the last 500 ns of the product runs from all replicates, to get representative structural ensembles at equilibrium, *via* the Jarvis-Patrick method (76) built in Gromacs. A structure is assigned to a cluster when this structure is a neighbor (*i.e.*, the top ten closest structures based on RMSD, a distance metric) of other structures in the same cluster, and they share at least three common neighbors. The cluster with the highest number of structures is considered the most populated cluster. For each cluster, the central structure of a cluster, defined by the smallest average RMSD from all other structures of the cluster, is selected as the representative structure of that cluster. All the representative structures in each cluster are visualized in VMD, but only the central structure from the most populated cluster is highlighted, and the rest are annotated as the background in structural ensemble visualizations. We also analyze hydrogen bonding, protein-solvent interaction energies and RMSF per residue at equilibrium using in-house and Gromacs scripts. We conduct secondary structure analysis at equilibrium stages using the edited DSSP package version 4.0 (77, 78). We perform dihedral angle analysis by plotting Ramachandran plots using the MDAnalysis Python library (79, 80, 81). We use in-house scripts to characterize hydration effects. We define hydrophilic hydration and hydrophobic hydration as follows: hydrophilic hydration occurs when water forms hydrogen bonds with hydrophilic atoms (oxygen, nitrogen, and hydrogen), and it most commonly occurs within the first two hydration layers (the first hydration layer is considered to be ∼ 2 Å, and the second one is ≤ 3.2 Å, as defined by the water radial distribution function data in Fig. S2), where the hydration layer is defined as the distance between water and protein, *e.g.*, water H to protein acceptor and *vice versa* for the first hydration layer. We refer to water forming protein–water H-bonds (hydrophilic local environment) as hydrophilic water. Hydrophobic hydration, by contrast, refers to water surrounding hydrophobic atoms (carbon or sulfur) and not forming protein-water H-bonds, which takes place in the third hydration layer (∼4 Å, based on RDF in Fig. S2). We refer to water in a hydrophobic local environment as hydrophobic water. We define local and global hydration as follows: local hydration refers to hydration surrounding single residues, in our case, Pro, Hyp, or Mop, while global hydration refers to hydration surrounding the entire models, including the full-length motif models, D18 models or tropoelastin molecule. The statistical significance of analysis results is examined by the SciPy Python library and is indicated in all figures as, ∗*p* < 0.05, ∗∗*p* < 0.01, ∗∗∗*p* < 0.001 (82).

## Data availability

All data generated or analyzed during this study are available from the corresponding author on reasonable request.

## Supporting information

This article contains supporting information. Supporting Information is available from the authors: Document S1. Figures S1-S12 and Tables S1 and S2.^8^, ^55^, ^64^, ^68^, ^83^, ^84^

## Conflict of interest

Anna Tarakanova reports financial support was provided by University of Connecticut. Anna Tarakanova reports a relationship with University of Connecticut that includes. The other authors declare that they have no conflicts of interest with the contents of this article.
