# Supplementary material for: Functions of prolyl hydroxylation in elastin
Source: J Biol Chem. 2026 Feb 26;302(4):111323. doi: 10.1016/j.jbc.2026.111323 (PMC13053751; doi:10.1016/j.jbc.2026.111323)
Supplement: Figures S1-S12 and Tables S1 and S2 [file mmc1.docx]

**Supplementary Information**

**Functions of Prolyl Hydroxylation in Elastin**

*Chengeng Yang^1^, Christian E. H. Schmelzer^2,3^, Anna Tarakanova^1,4,*^*

^1^Department of Biomedical Engineering, University of Connecticut

Storrs, CT, 06269, United States

191 Auditorium Road, Unit 3139, Storrs, CT, 06269, United States

^2^Department of Biological and Macromolecular Materials, Fraunhofer Institute for Microstructure of Materials and Systems IMWS, 06120 Halle (Saale), Germany

^3^Institute of Pharmacy, Faculty of Natural Sciences I, Martin Luther University Halle-Wittenberg, 06120 Halle (Saale), Germany

^4^School of Mechanical, Aerospace, and Manufacturing Engineering, University of Connecticut

Storrs, CT, 06269, United States

191 Auditorium Road, Unit 3139, Storrs, CT, 06269, United States

Corresponding author: Dr. Anna Tarakanova

*E-mail: anna.tarakanova@uconn.edu

**Secondary structure patterns in Hyp-containing models**

Despite apparent higher content of PPII helix, defined by dihedral angle pairs (ϕ, ψ) = (−75°, +145°) in Hyp-containing models of elastin-like peptides (ELPs) in experimental studies^1^, we did not detect such preference neither in our domain 18 models **(Figure S9B)** nor the full-length tropoelastin models **(Figure S9C)**. Reports in the literature have revealed a difference in PPII prevalence for different force fields. AMBER ff99SB-disp force field was deemed better than CHARMM36m ^2^ in detecting PPII helix in a previous work^3^. To compare, we benchmark the D18-1 model using the AMBER ff99SB-disp force field^4^, and calculate the detected secondary structure in **Figure S11**. Interestingly, even if the AMBER ff99SB-disp force field samples 1% more of PPII helix than the CHARMM36m force field, larger differences are found in turn and coil content. Based on these benchmarks, as the force field has minor effects on PPII content, we retain the use of CHARMM36m force field in our models, for consistency with our previous work.

Another possibility for this discrepancy with experimental findings may be scaling effects. In particular, aggregation of individual ELP molecules in experimental studies may lead to a higher propensity for PPII helix. In our simulations, only a single molecule is considered per model; no intermolecular protein-protein interactions are considered explicitly. We expect that future work can be focused on multimer aggregate simulations that may be more sensitive to force field selection.

Table S1. Mass spectrometry data of the content of Hyp in human elastin samples. Pro, proline; Hyp, hydroxyproline; IF2, human elastin isoform 2.

| **Name** | **Tissue** | **Species** | **# of Hyp** | **# of Pro** | **Hyp/ (Hyp+Pro) %** | **# of Hyp calculated in IF2** | **References** |
| --- | --- | --- | --- | --- | --- | --- | --- |
| HSAS 4544 | Aorta | Human | 28.7 | 120.8 | 19.198 | 16.51 | Hedtke et al., FEBS 2019 ^5^ |
| HSAS 4544 | Aorta | Human | 14.1 | 119.1 | 10.564 | 9.09 | Hedtke et al., FEBS 2019 ^5^ |
| HSAS 4543 | Skin | Human | 12.8 | 117.2 | 9.823 | 8.45 | Hedtke et al., FEBS 2019 ^5^ |
| HS3762 | Aorta | Human | 7.9 | 123.0 | 6.064 | 5.22 | Schmelzer et al., FASEB J 2019 ^6^ |


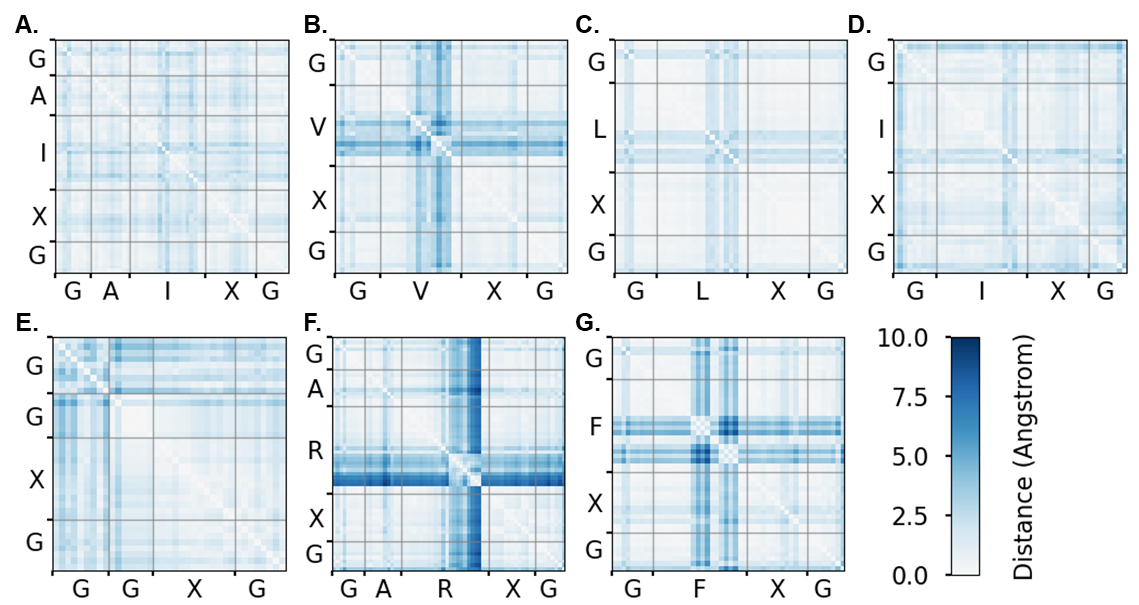


Figure S1. Heat maps that display absolute differences of the intramolecular atom-wise distance distributions between the Non-Hyp ensembles and Hyp-modified ensembles: (A) GAIPG vs. GAIHypG, (B) GVPG vs. GVHypG, (C) GLPG vs. GLHypG, (D) GIPG vs. GIHypG, (E) GGPG vs. GGHypG, (F) GARPG vs. GARHypG, and (G) GFPG vs. GFHypG.


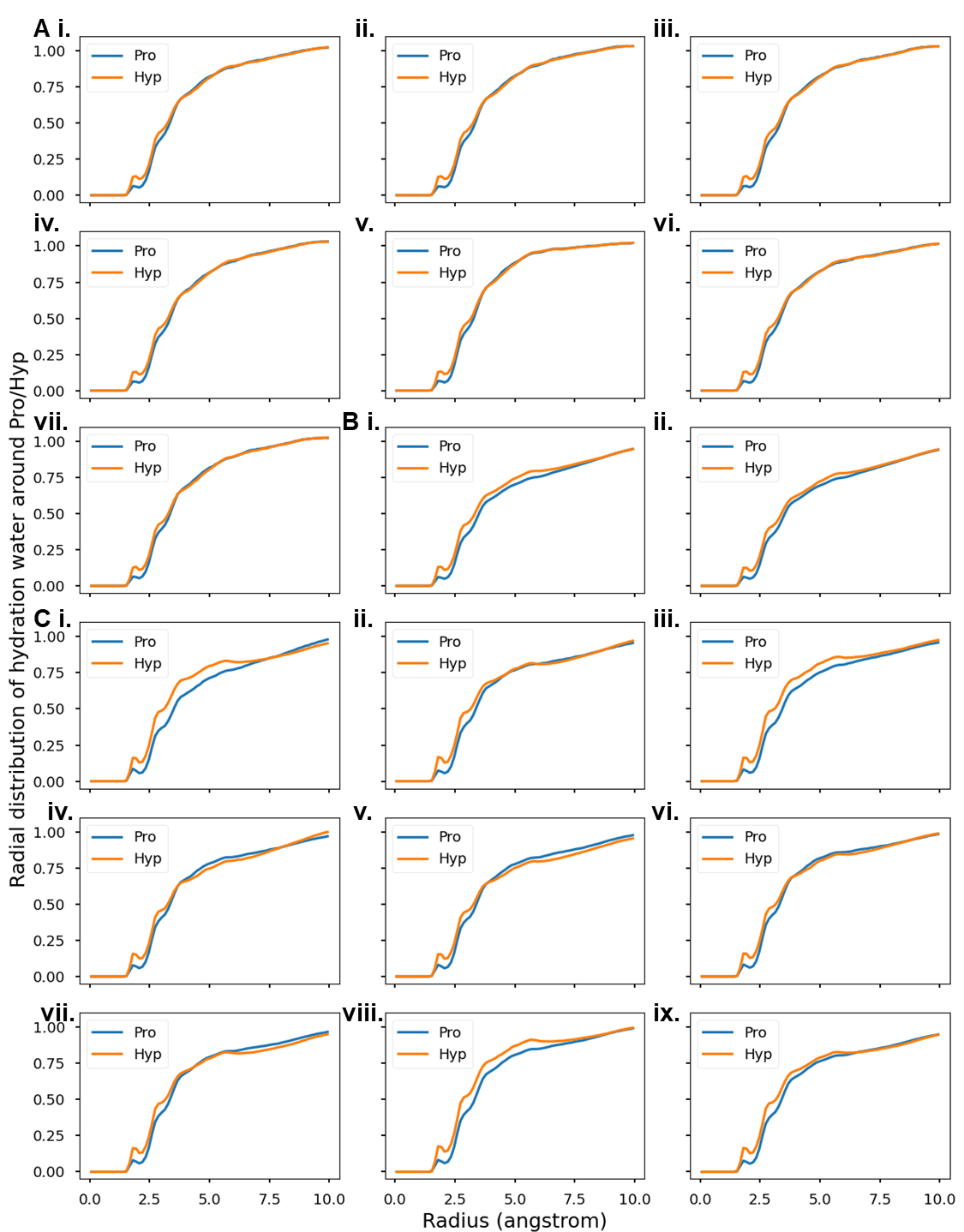


Figure S2. Water radial distribution function (RDF) of Proline vs. Hydroxyproline.

1. Local radial distribution function (RDF) of Proline vs. Hydroxyproline in the motif models: (i) GAIPG vs. GAIHypG, (ii) GVPG vs. GVHypG, (iii) GLPG vs. GLHypG, (iv) GIPG vs. GIHypG, (v) GGPG vs. GGHypG, (vi) GARPG vs. GARHypG, and (vii) GFPG vs. GFHypG.
2. Local radial distribution function (RDF) of Proline vs. Hydroxyproline in the domain 18 models: (i) D18-1 model and (ii) D18-2 model.
3. Local radial distribution function (RDF) of Proline vs. Hydroxyproline in the full-length tropoelastin models, (i) Non-Hyp vs. 5hyp-1, (ii) Non-Hyp vs. 5hyp-2, (iii) Non-Hyp vs. 8hyp-1, (iv) Non-Hyp vs. 8hyp-2, (v) Non-Hyp vs. 9hyp-1, (vi) Non-Hyp vs. 9hyp-2, (vii) Non-Hyp vs. 17hyp-1, (viii) Non-Hyp vs. 17hyp-2, (ix) Non-Hyp vs. 20hyp (overhydroxylation).


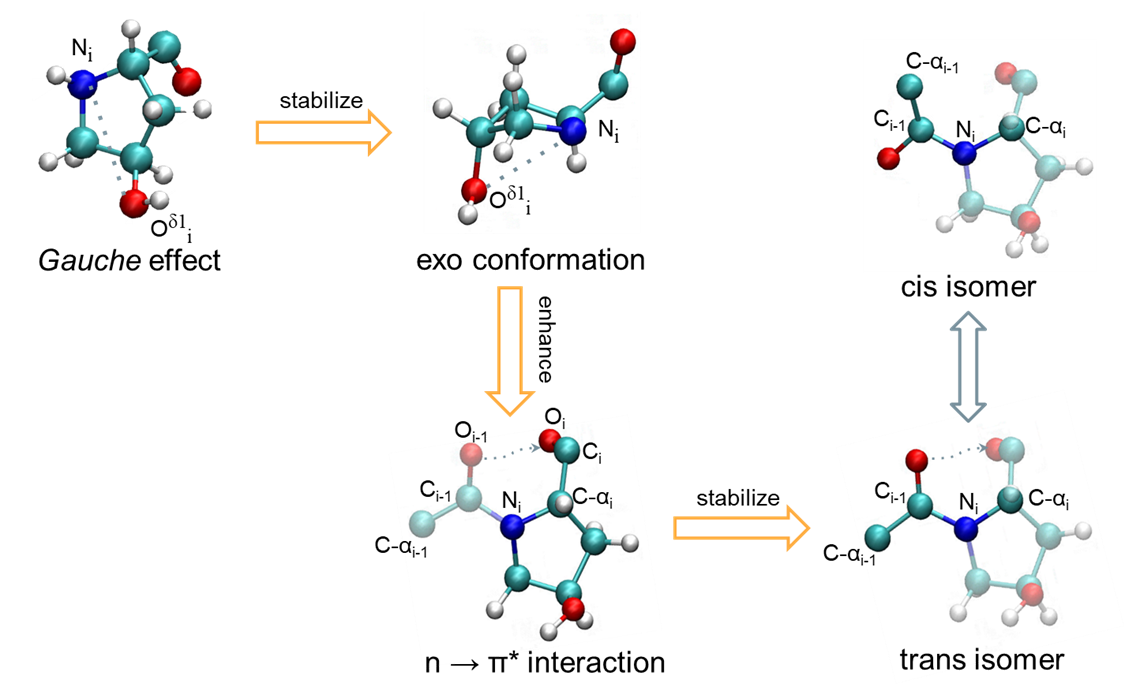


Figure S3. Proposed stereroelectronic effects on Hyp.


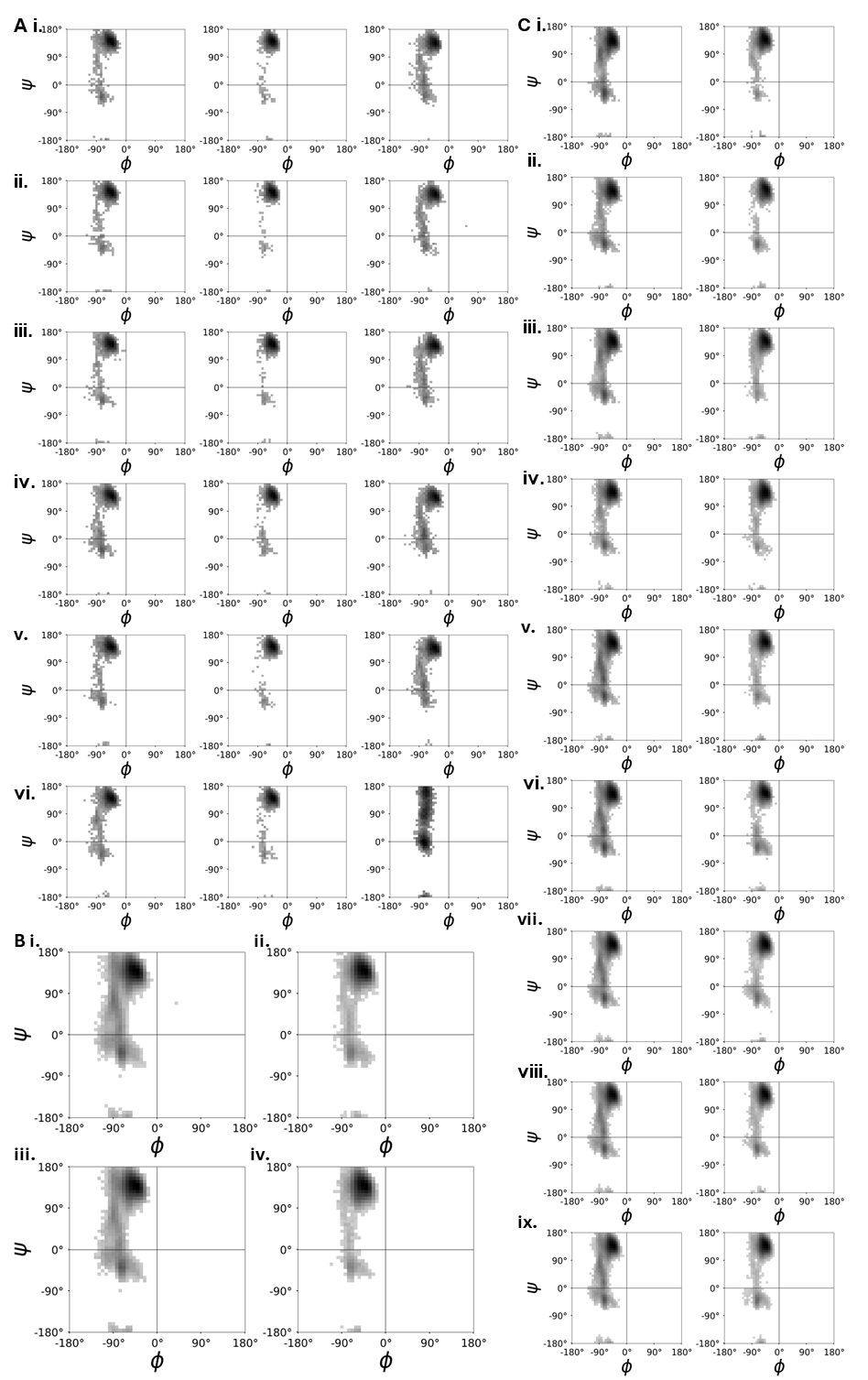


Figure S4. Main chain conformations of proline vs. its modification(s), showing the mainchain conformations.

1. Ramachandran plots of proline vs. hydroxyproline vs. methoxyproline in the motif models: (i) GVPG vs. GVHypG vs. GVMopG, (ii) GLPG vs. GLHypG vs. GLMopG, (iii) GIPG vs. GIHypG vs. GIMopG, (iv) GGPG vs. GGHypG vs. GGMopG, (v) GARPG vs. GARHypG vs. GARMopG, and (vi) GFPG vs. GFHypG vs. GFMopG.
2. Ramachandran plots of proline vs. hydroxyproline in the domain 18 models: (i) D18-1 model and (ii) D18-2 model.
3. Ramachandran plots of proline vs. hydroxyproline in the full-length tropoelastin models, (i) Non-Hyp vs. 5hyp-1, (ii) Non-Hyp vs. 5hyp-2, (iii) Non-Hyp vs. 8hyp-1, (iv) Non-Hyp vs. 8hyp-2, (v) Non-Hyp vs. 9hyp-1, (vi) Non-Hyp vs. 9hyp-2, (vii) Non-Hyp vs. 17hyp-1, (viii) Non-Hyp vs. 17hyp-2, (ix) Non-Hyp vs. 20hyp (overhydroxylation).


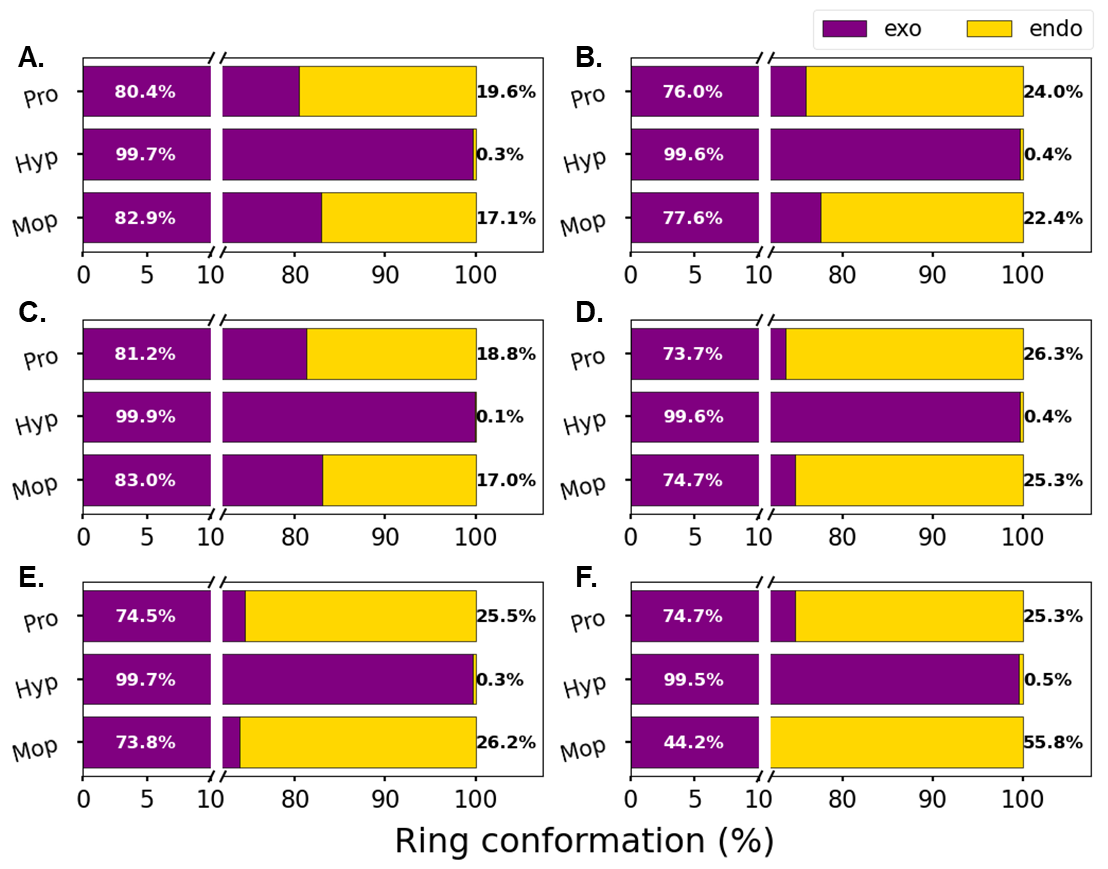


Figure S5. The preferred side chain conformations of proline vs. hydroxyproline vs. methoxyproline in the motif models: (A) GVPG vs. GVHypG vs. GVMopG, (B) GLPG vs. GLHypG vs. GLMopG, (C) GIPG vs. GIHypG vs. GIMopG, (D) GGPG vs. GGHypG vs. GGMopG, (E) GARPG vs. GARHypG vs. GARMopG, and (F) GFPG vs. GFHypG vs. GFMopG.


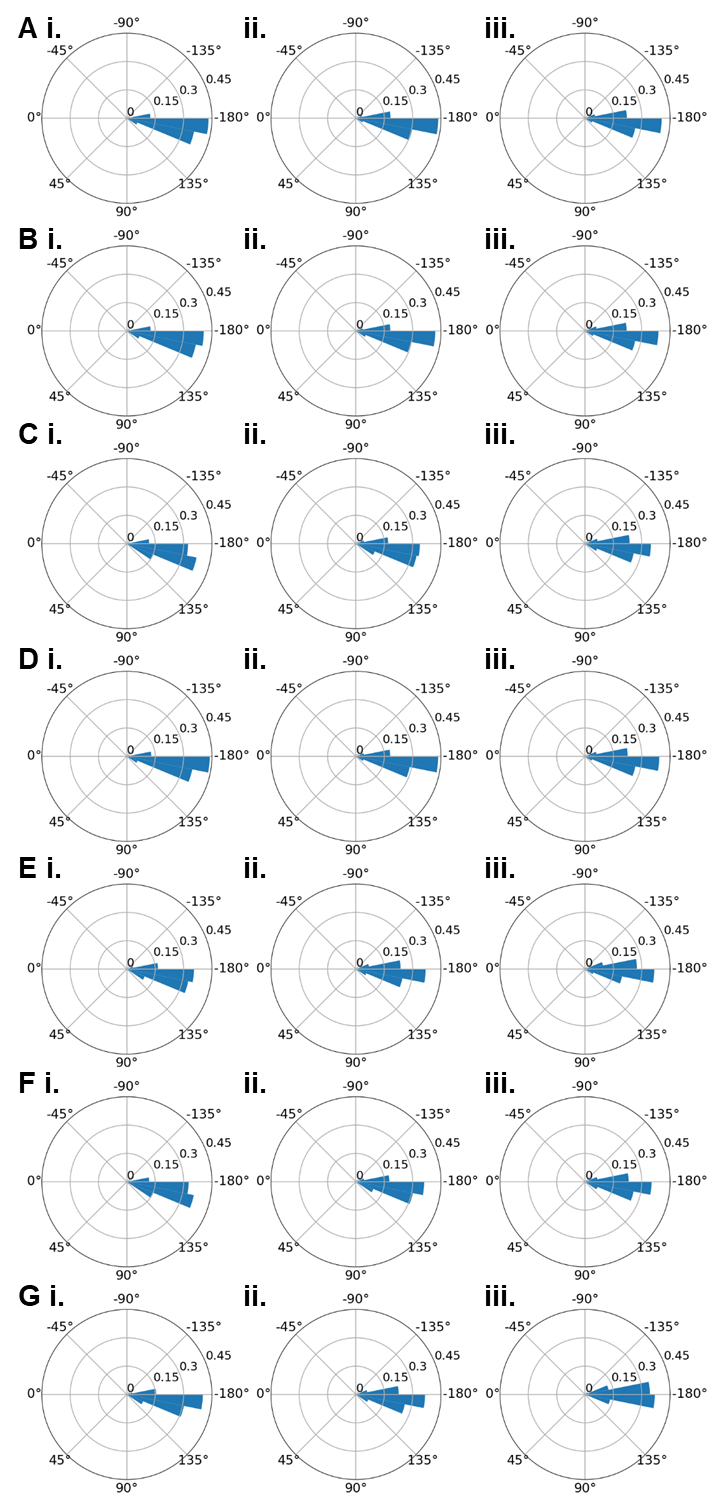


Figure S6. The polar histogram of dihedral angle ω of proline (Pro) vs. hydroxyproline (Hyp) vs. methoxyproline (Mop) in the motif models:

(A) (i) GAIPG vs. (ii) GAIHypG vs (iii) GAIMopG,

(B) (i) GVPG vs. (ii) GVHypG vs (iii) GVMopG,

(C) (i) GLPG vs. (ii) GLHypG vs (iii) GLMopG,

(D) (i) GIPG vs. (ii) GIHypG vs (iii) GIMopG,

(E) (i) GGPG vs. (ii) GGHypG vs (iii) GGMopG,

(F) (i) GARPG vs. (ii) GARHypG vs (iii) GARMopG,

(G) (i) GFPG vs. (ii) GFHypG vs (iii) GFMopG.


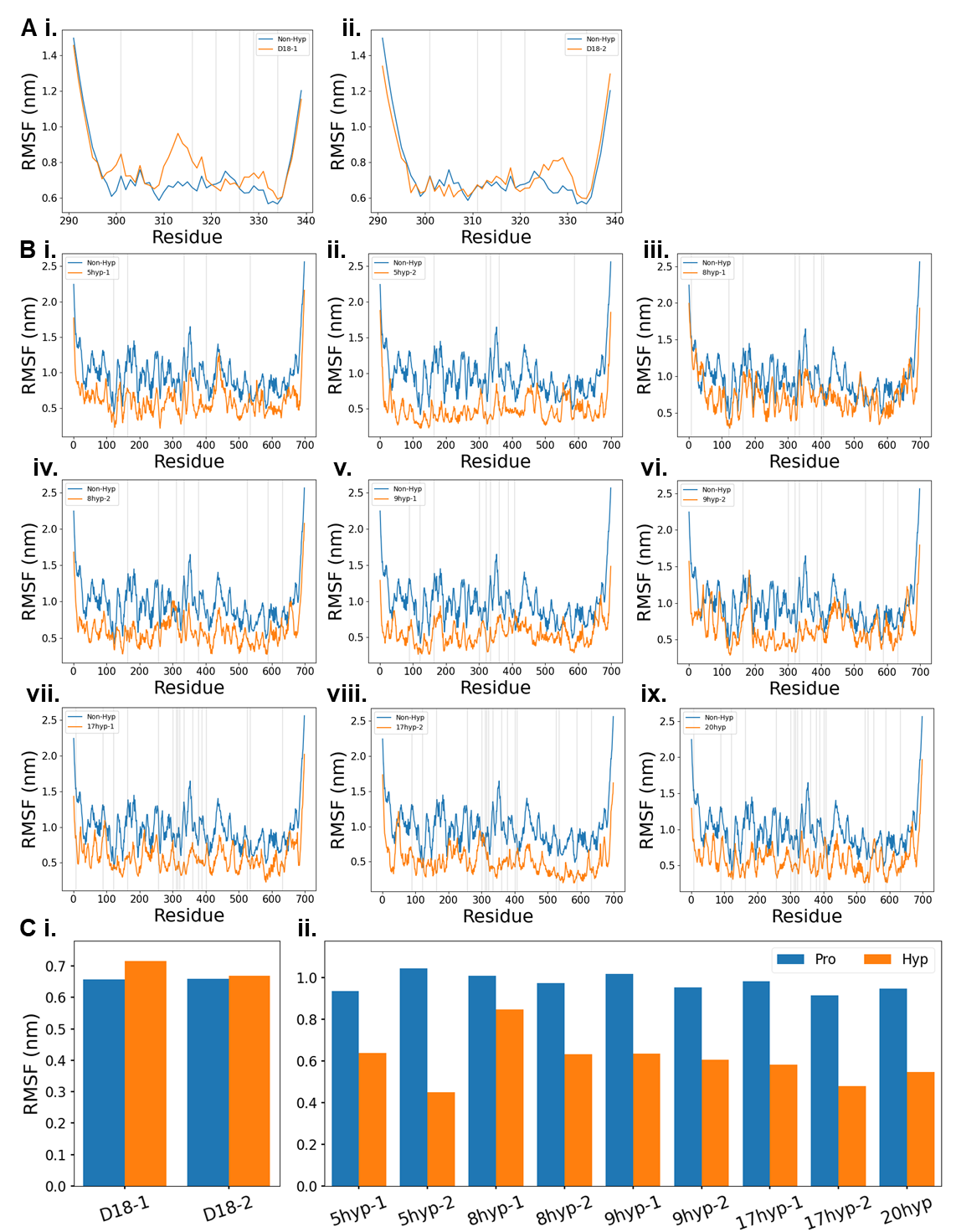


Figure S7. Local flexibility of domain 18 models and the full-length tropoelastin models.

1. Root mean square fluctuation (RMSF) of each residue in the domain 18 models: (i) D18-1 model and (ii) D18-2 model.
2. Root mean square fluctuation (RMSF) of each residue in the full-length tropoelastin models, (i) Non-Hyp vs. 5hyp-1, (ii) Non-Hyp vs. 5hyp-2, (iii) Non-Hyp vs. 8hyp-1, (iv) Non-Hyp vs. 8hyp-2, (v) Non-Hyp vs. 9hyp-1, (vi) Non-Hyp vs. 9hyp-2, (vii) Non-Hyp vs. 17hyp-1, (viii) Non-Hyp vs. 17hyp-2, (ix) Non-Hyp vs. 20hyp (overhydroxylation). The positions are hydroxyproline residues are marked by grey lines.
3. Root mean square fluctuation (RMSF) of Hyp residue vs. Pro residue in (i) the domain 18 models and (ii) the full-length tropoelastin models.


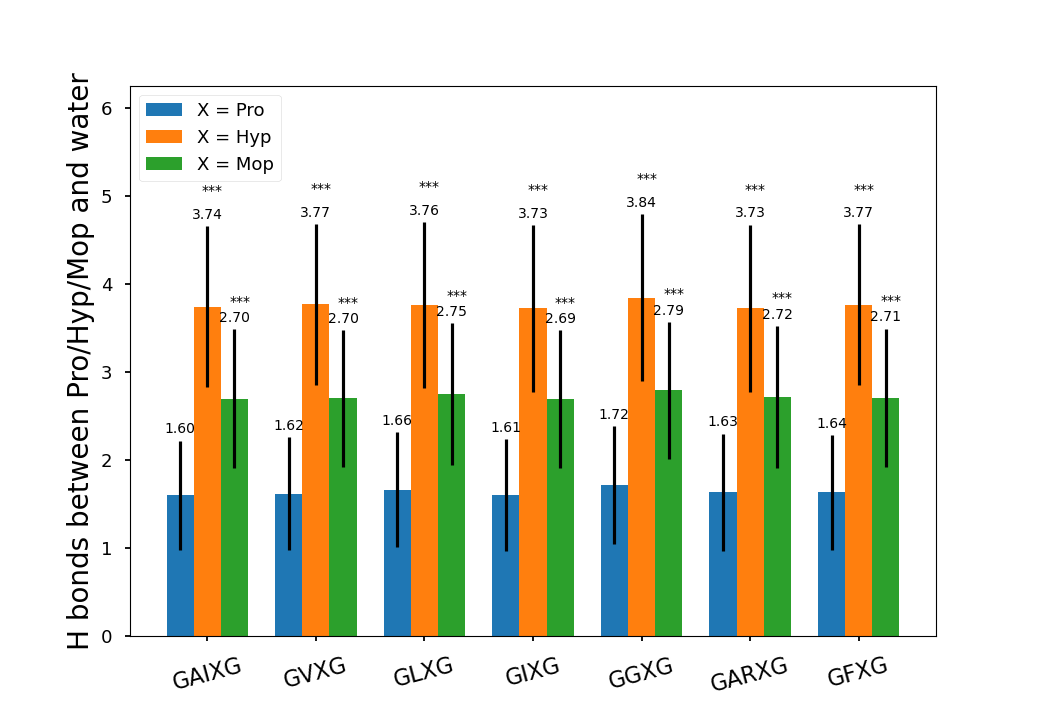


Figure S8. Local hydrogen bonding patterns between Proline vs. Hydroxyproline vs. Methoxyproline and water in the motif models. Significance is indicated in the figure captions as follows, * *p* < 0.05, ** *p* < 0.01, *** *p* < 0.001.


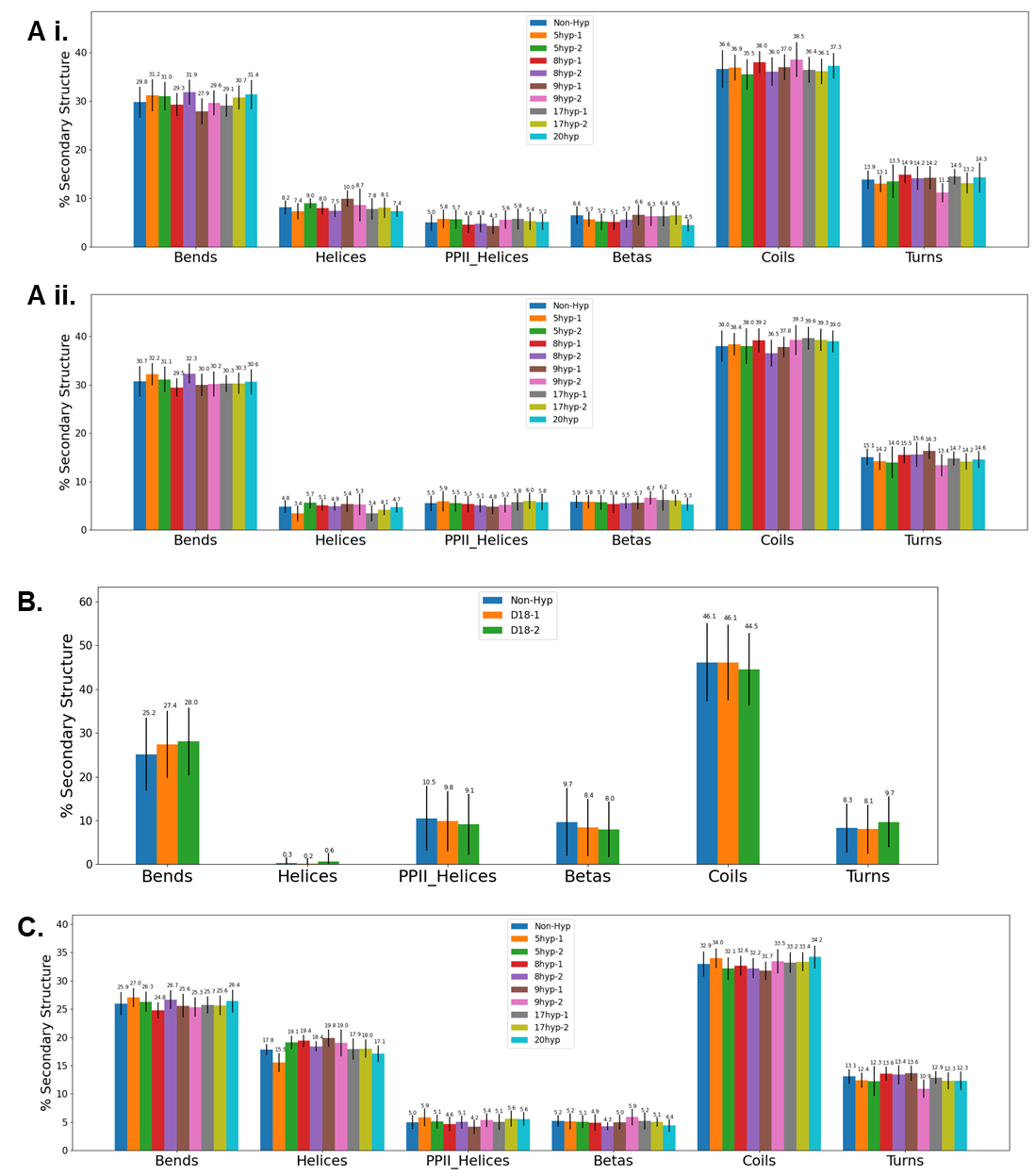


Figure S9. Local and global secondary structure distributions.

1. Local secondary structure distributions (i) of the PE targeting sites and (ii) of the NEP targeting sites. PE, pancreatic elastase. NEP, neprilysin.
2. Global secondary structure distributions in the domain 18 models.
3. Global secondary structure distributions in the full-length tropoelastin models.

Degrees of significance are indicated in the figure captions as follow, * *p* < 0.05, ** *p* < 0.01, *** *p* < 0.001.


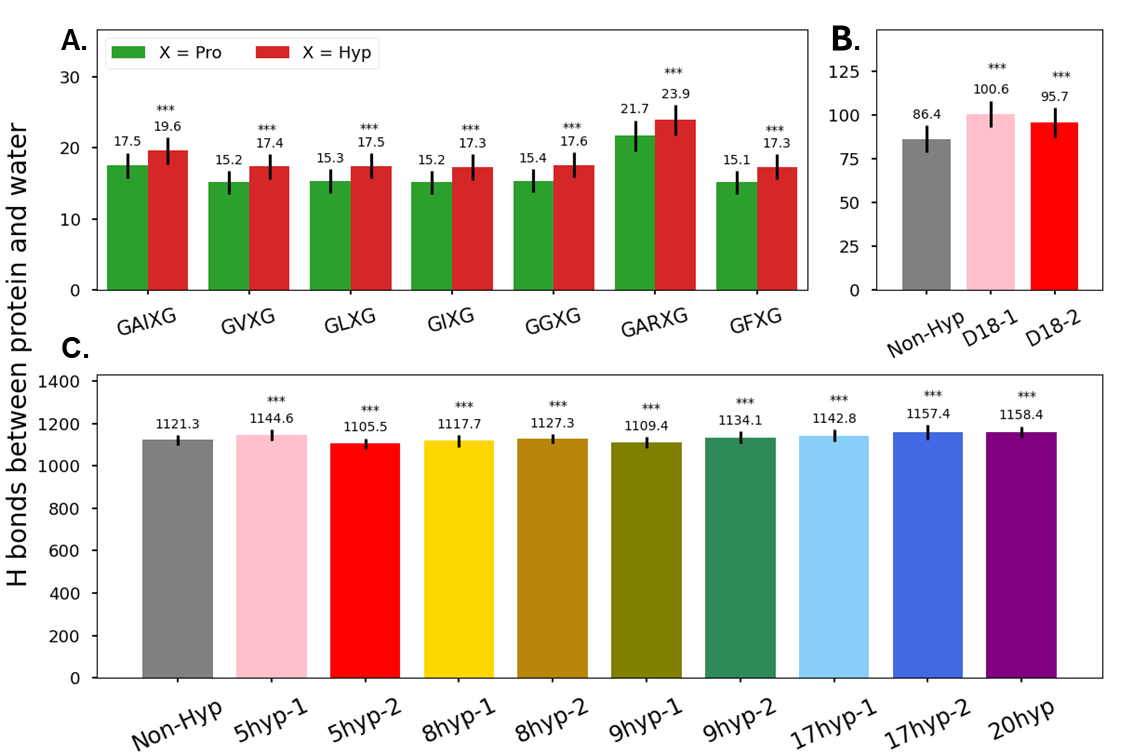


Figure S10. Global hydrogen bonding patterns between (A) the motif models and water, (B) the domain 18 models and water, (C) the full-length tropoelastin models and water. Significance is indicated in the figure captions as follows, * *p* < 0.05, ** *p* < 0.01, *** *p* < 0.001.

Table S2. Summary of statistical significance of difference in lysine contact maps between the non-hydroxylated full length tropoelastin model and hydroxylated full length tropoelastin models. Red: the distance values between lysine residue pairs in hydroxylated full length tropoelastin models are not statistically significantly higher than in the non-hydroxylated full length tropoelastin model. Green: the distance values between lysine residue pairs in hydroxylated full length tropoelastin models are statistically significantly higher *(*p < 0.05)* than in the non-hydroxylated full length tropoelastin model.

| **Cut-off (Å) / Models** | **5hyp-1** | **5hyp-2** | **8hyp-1** | **8hyp-2** | **9hyp-1** | **9hyp-2** | **17hyp-1** | **17hyp-2** | **20hyp** |
| --- | --- | --- | --- | --- | --- | --- | --- | --- | --- |
| **16** |  |  |  |  |  |  |  |  |  |
| **18** |  |  |  |  |  |  |  |  |  |
| **20** |  |  |  |  |  |  |  |  |  |
| **22** |  |  |  |  |  |  |  |  |  |
| **24** |  |  |  |  |  |  |  |  |  |
| **26** |  |  |  |  |  |  |  |  |  |
| **28** |  |  |  |  |  |  |  |  |  |
| **30** |  |  |  |  |  |  |  |  |  |


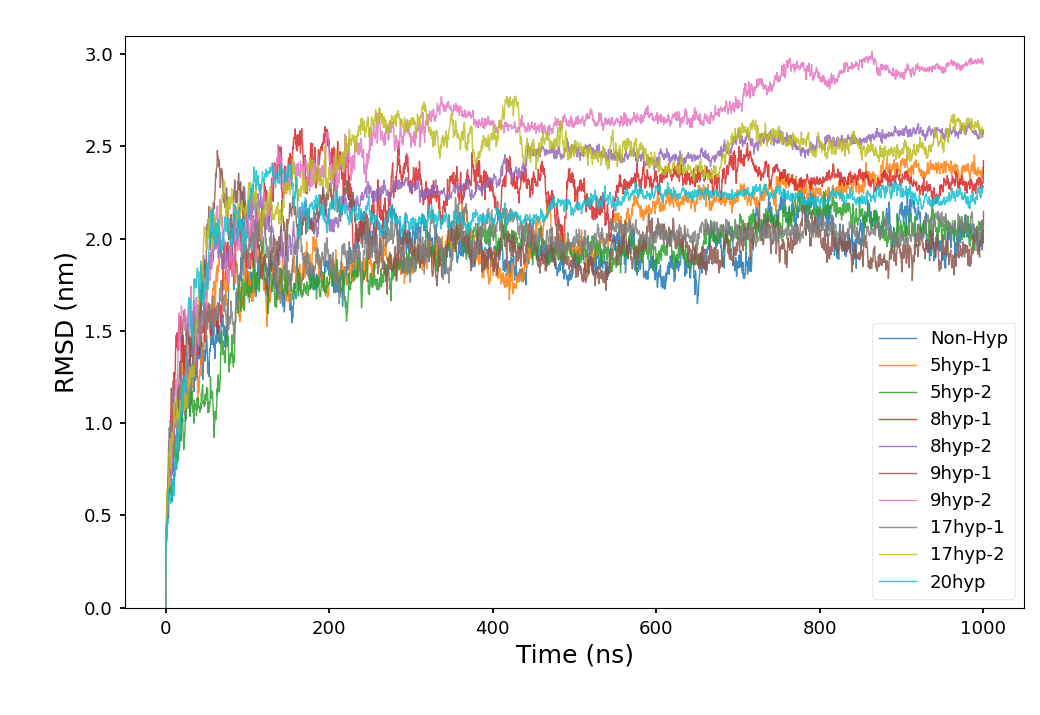


Figure 11. The root-mean square deviation (RMSD) of all full-length tropoelastin models, averaged from replicates.


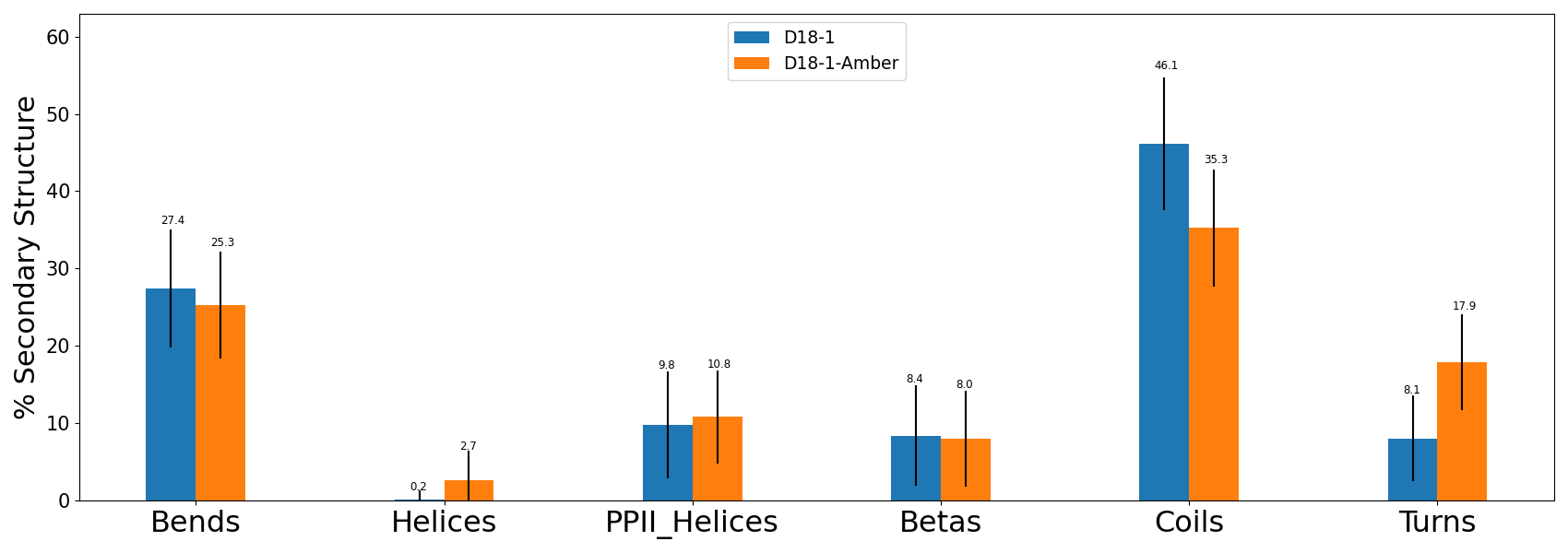


Figure S12. Comparison of CHARMM36m force field and AMBER99-disp force field for prediction of global secondary structure distributions in the domain 18 models. Significance is indicated in the figure captions as follows, * *p* < 0.05, ** *p* < 0.01, *** *p* < 0.001.

References

1. Bochicchio, B., Laurita, A., Heinz, A., Schmelzer, C.E., and Pepe, A. (2013). Investigating the role of (2S,4R)-4-hydroxyproline in elastin model peptides. Biomacromolecules *14*, 4278-4288. 10.1021/bm4011529.

2. Huang, J., Rauscher, S., Nawrocki, G., Ran, T., Feig, M., de Groot, B.L., Grubmuller, H., and MacKerell, A.D., Jr. (2017). CHARMM36m: an improved force field for folded and intrinsically disordered proteins. Nat Methods *14*, 71-73. 10.1038/nmeth.4067.

3. Jephthah, S., Pesce, F., Lindorff-Larsen, K., and Skepo, M. (2021). Force Field Effects in Simulations of Flexible Peptides with Varying Polyproline II Propensity. J Chem Theory Comput *17*, 6634-6646. 10.1021/acs.jctc.1c00408.

4. Robustelli, P., Piana, S., and Shaw, D.E. (2018). Developing a molecular dynamics force field for both folded and disordered protein states. Proc Natl Acad Sci U S A *115*, E4758-E4766. 10.1073/pnas.1800690115.

5. Hedtke, T., Schrader, C.U., Heinz, A., Hoehenwarter, W., Brinckmann, J., Groth, T., and Schmelzer, C.E.H. (2019). A comprehensive map of human elastin cross-linking during elastogenesis. FEBS J *286*, 3594-3610. 10.1111/febs.14929.

6. Schmelzer, C.E.H., Hedtke, T., and Heinz, A. (2020). Unique molecular networks: Formation and role of elastin cross-links. IUBMB Life *72*, 842-854. 10.1002/iub.2213.
